# Evolutionarily novel genes are expressed in transgenic fish tumors and their orthologs are involved in development of progressive traits in humans

**DOI:** 10.1186/s13027-019-0262-5

**Published:** 2019-12-05

**Authors:** E. A. Matyunina, A. V. Emelyanov, T. V. Kurbatova, A. A. Makashov, I. V. Mizgirev, A. P. Kozlov

**Affiliations:** 1 Research Institute of Ultra-Pure Biologicals, Ministry of Public Health of the Russian Federation, St.-Petersburg, Russia; 2 Peter the Great Saint-Petersburg Polytechnic University (SPbPU), St.-Petersburg, Russia; 3 The Biomedical Center (BMC), St.-Petersburg, Russia; 4 Institute for Research on Cancer and Aging (IRCAN), Nice, France; 5 Petrov Research Institute of Oncology, St.-Petersburg, Russia; 6 Vavilov Institute of General Genetics, Russian Academy of Sciences, Moscow, Russia

**Keywords:** Cancer, Evolution, Gene expression, Tumor specific expression, Evolutionarily novel

## Abstract

**Abstract:**

Earlier we suggested a new hypothesis of the possible evolutionary role of hereditary tumors (Kozlov, Evolution by tumor Neofunctionalization, 2014), and described a new class of genes – tumor specifically expressed, evolutionarily novel (*TSEEN*) genes - that are predicted by this hypothesis (Kozlov, Infect Agents Cancer 11:34, 2016). In this paper we studied evolutionarily novel genes expressed in fish tumors after regression, as a model of evolving organs. As evolutionarily novel genes may not yet have organismal functions, we studied the acquisition of new gene functions by comparing fish evolutionarily novel genes with their human orthologs. We found that many genes involved in development of progressive traits in humans (lung, mammary gland, placenta, ventricular septum, etc.) originated in fish and are expressed in fish tumors and tumors after regression. These findings support a possible evolutionary role of hereditary tumors, and in particular the hypothesis of evolution by tumor neofunctionalization.

**Research highlights:**

Earlier we described a new class of genes that are tumor-specifically expressed and evolutionarily novel (*TSEEN*). As the functions of *TSEEN* genes are often uncertain, we decided to study *TSEEN* genes of fishes so that we could trace the appearance of their new functions in higher vertebrates. We found that many human genes which are involved in development of progressive traits (placenta development, mammary gland and lung development etc.,) originated in fishes and are expressed in fish tumors.

## Background

We are interested in the possible role of tumors in evolution. In previous publications [22–27] the hypothesis of the possible evolutionary role of hereditary tumors was formulated. According to this hypothesis, hereditary tumors at earlier stages of progression, or benign tumors, were the source of extra cell masses which could be used during the evolution of multicellular organisms for the expression of evolutionarily novel genes, for the origin of new differentiated cell types with novel functions, and for building new structures that constitute evolutionary innovations and morphological novelties. Hereditary tumors could play an evolutionary role by providing conditions (space and resources) for the expression of genes that have newly-arisen in the germline. As a result of the expression of such novel genes, tumor cells may acquire new functions and differentiate in new directions, which might in turn lead to the origin of new cell types, tissues and organs [26].

This hypothesis makes several nontrivial predictions. One prediction is that tumors could be beneficial to the organism by performing new functional roles. This prediction was addressed in previous work [26, 29], where it was shown that the «hoods» of some varieties of gold fishes such as Lionhead, Oranda, etc. are benign tumors. These tumors have been selected by breeders for hundreds of years until they eventually formed new organ, the «hood». The origin of simbiovilly in voles is the result of natural selection of early papillomatosis (Vorontsov, 2003), and the origin of macromelanophores in swordtails is the result of sexual selection [18]. These examples were discussed in detail in [26]. They support the prediction about the possibility of selection of hereditary tumors for new organismal functions.

Another prediction of the hypothesis is that evolutionarily young and novel genes should often be specifically (or preferentially) expressed in tumors. This prediction was verified in a number of papers from our laboratory ([6, 20, 28, 30, 40–42, 44]; Dobyunin et al., 2013 [27];). We have described several evolutionarily young and novel genes with tumor-predominant or tumor-specific expression in humans, and even the evolutionary novelty of an entire class of genes – cancer/testis genes – which includes evolutionary young and novel genes expressed predominantly in tumors (reviewed in [27]).

We suggested to call such genes tumor specifically expressed, evolutionarily novel (*TSEEN*) genes [25–27].

The functional role of evolutionarily novel genes is often uncertain. Therefore, we were further interested in studying evolutionarily novel genes of fishes, so that we could trace the evolutionary trajectory of the appearance of their new functions in higher vertebrates. Our hypothesis predicts that some fish *TSEEN* genes should have acquired functions that determine progressive traits during evolution in higher vertebrates including humans. In the present study, we have used the transgenic inducible hepatoma model in zebrafish described earlier [34], because we suppose that transgenic tumors, after regression, may be an approximation to an evolving organ. So, we studied evolutionarily novel genes in fish, that are expressed both in tumors and in tumors after regression.

## Materials and methods

### Transgenic inducible hepatoma model

The *kras*^*V12*^-induced tumor progression was conducted on 100 transgenic zebrafish fishes maintained in water containing 2 μM mifepristone. Fishes were treated at age of reproduction, more than 2 month pf (post fertilization), between 4 and 6 month old. All experimental zebrafish can be described as siblings, as progeny of single pair of parents. Gross morphological and histological analyses were weekly performed on 15 randomly selected fishes to monitor tumor development. These analyses showed robust development of hepatocellular carcinoma within 4 weeks of induction. Observation of tumor development and hepatocellular carcinoma staging of tumorigenesis was conducted as described [34].

For tumor regression the group of 15 fishes with hepatocellular carcinoma stage (according to [34]) was transferred to mifepristone-free water. Gross observation revealed shrinkage of tumor and dissappearence of GFP. Notably, complete tumor regression with scarred fibrosis of the former tumor tissue was observed after 4 weeks of mifepristone withdrawal.

Liver tumors from mifipristone induced transgenic fishes with hepatocellular carcinoma, livers after tumor regression (from fishes after mifepristone withdrawal) and normal livers from non-induced transgenic fishes were pooled separately and collected for RNA isolation and sequencing.

### RNA sequencing and sequence data analysis

Total RNA was extracted using TRIzol Reagent (Invitrogen, USA) and treated with DNase I to remove genomic DNA contamination. mRNA was purified using Dynabeads Oligo (dT) EcoP (Invitrogen) and subjected to cDNA synthesis. Resultant cDNA was digested by NlaIII and EcoP15I to result in a 27 nucleotides cDNA tag between the two sequencing adapters. 3′ RNA-SAGE (serial analysis of gene expression) sequencing was performed on ABI SOLiD platform by Mission Biotech (Taiwan) according to manufacturer’s protocol and 10–23 million reads were generated from each sample (Additional files 1, 2, 3). The tags were mapped to the NCBI RefSeq (Reference Sequence) [36] mRNA database for zebrafish with a criterion of maximum 2 nucleotide mismatches.

All RNA-Seq data were submitted to Gene Expression Omnibus database [16] and are accessible through GEO Series accession number GSE93965.

Tag counts for each transcript were normalized to TPM (transcripts per million) to facilitate comparison among different samples.

### Transcriptome coverage estimation

In order to estimate RNA-Seq sensitivity we chose several housekeeping genes with known expression level in normal liver. The list of these genes is presented in Additional file 4. Relative copy number value was studied for each these genes. For further transcriptome analysis we chose gluconeogenesis metabolic pathway (15 genes according to GO,0006094; GO:0006111; GO:0035948; GO:0045722; GO:0045721).

### Selection of genes activated in tumors and expressed after regression

After RNA deep sequencing the lists of transcripts in control, tumor and regressed tumor samples (the original data after raw Blast from the company) were analyzed.

We manually selected genes which were expressed in liver carcinomas and in liver after regression of tumors but not in normal liver.

### The search of orthologs

*Danio rerio* genome was retrieved from *Danio rerio* genome sequencing project (GRCz10). GRCz10 (Genome Reference Consortium Zebrafish Build 10, INSDC Assembly GCA_000002035.3, Sep 2014).

For the search of orthologs we chose the following genomes: lamprey (*Petromyzon marinus*, Pmarinus_7.0); spotted gar (LepOcu1, *Lepisosteus oculatus*), atlantic cod (gadMor1, *Gadus morhua*); clawed frog (JGI 4.2, *Xenopus tropicalis*); human (GRCh38, *Homo sapiens*).

These genomes were downloaded via ensembl ftp browser, the command line used are presented in the Supplementary text.

For the search of orthologs we used BLAST command line applications developed at the National Center for Biotechnology Information (NCBI). We required to run BLAST locally and to support automatic resolution of sequence identifiers [10]. Documentation about this procedure can be found in ftp://ftp.ncbi.nlm.nih.gov/blast/db/README.

The blastx, and psiblast were considered search applications, as they execute the BLAST search [4].

We ran NCBI’s blastx alignment search for all nucleotide sequences of the coding regions of our genes from the sample against Nucleotide database of genomes chosen above. The Nucleotide database is a collection of sequences from several sources, including GenBank, RefSeq, TPA and PDB.

We ran NCBI’s psiblast comparisons of all proteins encoded by annotated genes of our sample against all the proteins encoded by genes annotated in any chosen genome, with a E-value threshold of 1 × 10 ^− 3^.

All of the blast algorithms were used via python scripts (version Python 3.4.6 Available at http://www.python.org) using Biopython modules [14] for running BLAST locally.

For any further consideration, we also required the coverage of at least 25% of any of the protein sequences in the alignments.

We imported the output of blastx and psiblast into a MySQL database where we filtered the matches to sequences with alignment coverage more then 25% and E-value below the chosen cut off (1 × 10 ^− 3^). The final E-value 1 × 10^− 3^ cutoff value was based on our analysis of the single genes from our sample as a compromise between specificity and sensitivity. Orthology data from EnsemblCompara resource [48] were loaded into the same MySQL database. The final results of the search of orthologs were annotated via SQL queries by joining tables using ensembl Gene ID, Additional file 23.

### OMA («orthologous matrix»)

As an alternative method for seeking orthologs we used OMA (Orthologous MAtrix) for large-scale orthology inference [43]. The advantages of this approach are the use of evolutionary distances instead of BLAST scores, consideration of distance inference uncertainty, including many-to-many orthologous relations, and accounting for differential gene losses. In our search we used the following genomes: elephant shark (Callorhinchus_milii-6.1.3, *Callorhinchus milii*), common carp (ASM127010v1, *Cyprinus carpio*), tunicate (KH, *Ciona intestinalis*), atlantic herring (ASM96633v1, *Clupea harengus*), coelacanth (latCha1, *Latimeria chalumnae*), lamprey (*Petromyzon marinus*, Pmarinus_7.0), Hagfish (*Eptatretus burger*, Eburgeri_3.2 (GCA_900186335.2)), spotted gar (LepOcu1, *Lepisosteus oculatus*), red-bellied piranha (Pygocentrus_nattereri-1.0.2, *Pygocentrus nattereri*), whale shark (ASM164234v2, *Rhincodon typus*); atlantic salmon (ASM23337v1, *Salmo salar*), asian bonytongue (ASM162426v1, *Scleropages formosus*), chimpanzee (Pan_tro 3.0, *Pan troglodytes*), orangutan (Susie_PABv2, *Pongo abelii*), platypus (Ornithorhynchus_anatinus-5.0.1, *Ornithorhynchus anatinus*), opossum (MonDom5, *Monodelphis domestica*), mouse (GRCm38.p6, *Mus musculus*), rat (Rnor_6.0, *Rattus norvegicus*), sea urchin (Spur_4.2, *Strongylocentrotus purpuratus*), clawed frog (Xenopus_tropicalis_v9.1, *Xenopus tropicalis*), yeast (Sc_YJM993_v1, *Saccharomyces cerevisiae*) and human (GRCh38, *Homo sapiens*).

### cDNA panels

The panels from various normal human tissues containing a set of normalized single-strand cDNA, produced from poly(A) + RNA were obtained from Clontech, USA. We used the following panels: Human MTC™ Panel I (Cat. no. 636742), Human MTC™ Panel 2 (Cat. no. 637643), Human Fetal MTC™ Panel (Cat. no. 636747). According to the manufacturer’s information, the panels were free from genomic DNA and were normalized to expression levels of four house-keeping genes. According to the manufacturer’s information, each cDNA sample comes from a pool of tissue samples obtained from donors of different age and sex, with 2–550 donors in each pool, and the fetal tissue samples were obtained from spontaneously aborted fetuses at 18 to 36 weeks of gestational age. The relevant ethics statement is available from manufacturer’s website: Takara Bio Inc., USA.

A cDNA panel from human tumors containing a total of 10 of cDNA samples were obtained from BioChain Instutute, USA (Cat. nos. C1235035–10, C1235086, C1235090, C1235142, C1235152, C1235183–10, C1235188–10, C1235201–10, C1235248, C1235274). The samples were produced by the manufacturer from various human tumors obtained by surgerical resection. Each sample came from one patient and was histologically characterized. cDNA was produced from poly(A) + mRNA that was free from genomic DNA and normalized by b-actin gene expression level. The relevant ethics statement is available from manufacturer’s website: BioChain Institute Inc., USA.

### Zebrafish normal liver and cancers tissues

Zebrafish were maintained according to established protocols [50]. All experimental procedures with fishes, such as gross observation and sampling of materials were carried out in accordance with regulations of institutional ethical committee.

Each sample from zebrafish was histologically characterized. We used tissues of spontaneous hepatocellular carcinoma and spermatocytic seminoma, and pooled samples of normal liver from 4 to 5 fishes.

### RNA purification and cDNA synthesis for gene expression experiments

The total RNA from fish liver and tumor samples was extracted using TRIzol Reagent (Invitrogen, USA) following the standard protocol. The isolated RNA had an A_260/280_ ratio of ≥1.7 when diluted into distilled water. Ethidium bromide staining of RNA in agarose gels visualizes two predominant bands of small and large ribosomal RNA, a bands of low molecular mass RNA.

cDNA synthesis was performed from equal amounts of RNA using Revert Aid® First Strand cDNA Synthesis Kit (Thermo, USA) with random hexanucleotide, following the manufacturer guidelines. The obtained cDNA was stored at − 20 °C.

### PCR

The PCR mixture contained 2.5 μl of cDNA, PCR-buffer (67 mMTris-HCl, pH 8.9, 4 mM MgCl2, 16 mM (NH_4_)_2_SO4, 10 mM 2-mercaptoetanol), 200 μM dNTP, 1 unit of Taq DNA polymerase (Fermentas, Lithuania), and 10 pmol of forward and reverse primers in a total of 25-μl reaction. Amplification was performed in a thermal cycler C1000TM Thermal Cycler, Bio-Rad, USA.

All primers targeting zebrafish genes were chosen to cross exon-intron junctions in order to avoid amplifying genomic DNA. The following PCR conditions were used: 3 min at 95 °C; 35 cycles consisting of 30 s at 95 °C, 30 s at 58 °C, 30 s at 72 °C; and final elongation at 72 °C for 5 min. We used zebrafish *gapdh* gene primers as a positive control for gene expression. Primer sequences used for PCR and the expected size of amplicons are included in Additional file 7.

Primer sequences for experimental studies of expression of human orthologs of fish *TSEEN* genes in cDNA panels from human normal tissues and the expected size of amplicons are included in Table of Additional file 7. Amplification was performed with the following conditions: 3 min at 95 °C; 35 cycles consisting of 30 s at 95 °C, 30 s at 58 °C, 30 s at 72 °C; and final elongation at 72 °C for 5 min. We used human GAPDH gene primers as a positive control for gene expression; the following PCR conditions were used 3 min at 95 °C; 30 cycles consisting of 30 s at 95 °C, 30 s at 68 °C, 1 min at 72 °C; and final elongation at 72 °C for 5 min. The expected size of the *GAPDH* – specific product was 983 bp.

All PCR products were analyzed by electrophoresis in 1.8% agarose gel and detected by staining with ethidium bromide. The results of electrophoresis are presented as truncated images of gels.

### The study of gene expression in normal zebrafish tissues treated with mifepristone

In order to exclude the possible gene expression activation by mifepristone, 20 to 50 fishes were treated at 5 mkM of mifepristone for 5 days. In 5 day old fishes the liver is already developed.

Total RNA was isolated using the RNeasy Mini Kit (QIAGEN) and treated with DNase (QIAGEN) in accordance with the manufacturer’s instructions. 2 μg of total RNA was reverse transcribed using RevertAid First Strand cDNA Synthesis Kit (Thermo ScientificTM) and 50–70 ng cDNA were used in triplicate for qPCR. Quantitative real-time PCR reactions were performed with the SYBER Green (KAPA Biosystem) in MicroAmp Optical 96-well plates using a StepOnePlus System (Applied Biosystems). See Additional file 15 for a complete list of qRT-PCR primer sequences used in this study.

### Functional annotation

Functional annotation was accomplished with the help of the Gene Ontology tool [5]. GO annotation for genes from our samples were retrieved from EnsemblBiomart resource (Ensembl 89: May 2017) and stored in MySQL database. Several tables were created, depending on evolutionary novelty of genes and GO evidence codes (Additional files 9, 10, 11 and 12).

The Experimental Evidence codes are the following: inferred from experiment (EXP), inferred from direct assay (IDA), inferred from physical interaction (IPI), inferred from mutant phenotype (IMP), inferred from genetic interaction (IGI), inferred from expression pattern (IEP).

In order to estimate the GO enrichment of human orthologs of fish *TSEEN* genes GeneOntology enrichment analysis and visualization tool was used [33, 45]. As a background we used a list of all human genes.

## Results

After filtering against multiple hits for tagged sequences and re-annotation, Additional file 1 contains the list of 16,083 normal liver transcripts and their descriptions, Additional file 2– the list of 14,334 carcinoma transcripts and their description, Additional file 3– the list of 8812 transcripts expressed in liver after tumor regression. Transcriptome normalization was made by housekeeping gene transcripts abundance estimation, Additional file 4, based on the measured transcript levels of all genes involved in gluconeogenesis metabolic pathway (15 genes according to GO:0006094; GO:0006111; GO:0035948; GO:0045722; GO:0045721).

**Fig. 1:**
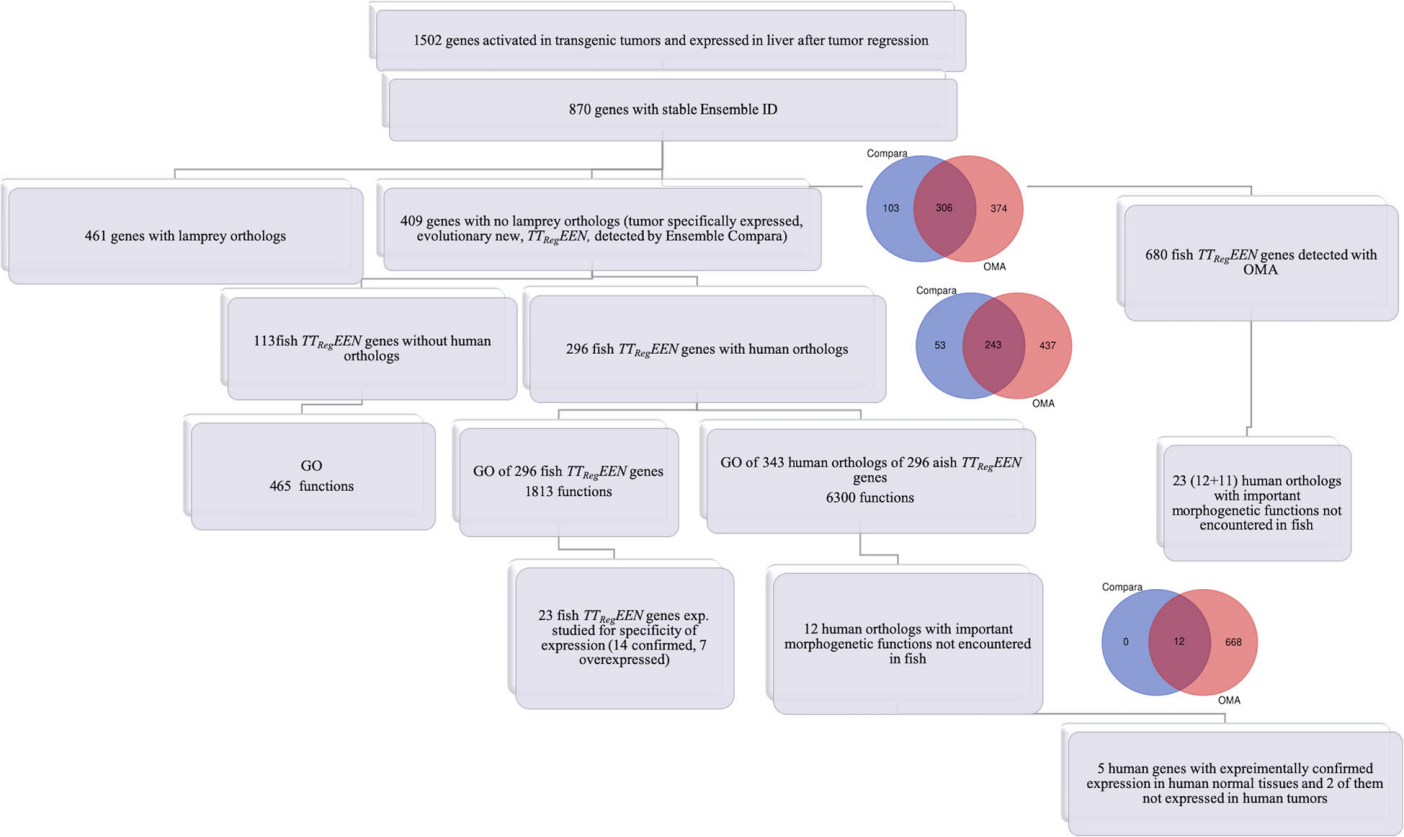
Flow diagram for the study of selected groups of zebrafish genes and their human orthologs

By comparison of the results of deep sequencing of RNA from normal liver, liver tumor and liver after tumor regression we manually selected a sample of 1502 genes which were activated in tumors and expressed after tumor regression, as described in materials and methods (Fig. 1 and Additional file 5).

From these 1502 genes, we selected 870 genes that had stable Ensembl gene IDs (Fig. 1). Among these 870 genes with the Ensembl gene IDs, 868 are protein coding, 1 is a polymorphic pseudogene and 1 is a lncRNA not present in control liver tissues (Additional file 6).

We sought orthologs for the set of 870 genes in 5 genomes of different species, which were selected relative to the phylogenetic position of zebrafish. We estimated the number of genes that have orthologs in the chosen genomes using three algorithms, based both on blast alignments and tree construction (cut off e-value < 10^− 3^ and matching > 25% of the total protein length) (Table 1). Among the 870 genes with Ensembl gene IDs, 461 had lamprey orthologs, and 409 had no lamprey orthologs ***(***Fig. 1***)***. We defined the 409 genes with no orthologs in genomes of lamprey as evolutionarily novel to fishes. These genes are tumor and tumor-after-regression expressed, evolutionarily novel (*TT*_*Rgr*_*EEN*) genes.

**Table 1:** The number of zebrafish genes activated in liver transgenic tumors and expressed in liver after tumor regression, which have orthologs in the genomes of different species found by different algorithms

Organism	Species	Genome	BLASTX	PsiBlast	Ensembl89: May 2017
			25%, eval< 10^− 3^	25%, eval< 10^− 3^	25%, conf = 1
Zebrafish	*Danio rerio*	GRCz10, INSDC Assembly GCA_000002035.3, Sep 2014	870	870	870
Lamprey	*Petromyzon marinus*	Pmarinus_7.0, Jan 2011	377	522	461
Spotted Gar	*Lepisosteus oculatus*	GRCz10, INSDC Assembly GCA_000002035.3, Sep 2014	659	–	725
Atlantic Cod	*Gadus morhua*	gadMor1, Jan 2010	580	593	632
Xenopus	*Xenopus tropicalis*	JGI 4.2, INSDC Assembly GCA_000004195.1, Nov 2009	599	–	644
Human	*Homo sapiens*	GRCh38.p3, INSDC Assembly GCA_000001405.18, Dec 2013	624	593	689

As shown in Table 1, the 870 zebrafish genes have a considerable number of orthologs in the genomes of the selected species. The results obtained by different algorithms are comparable. For subsequent work, we used results obtained by Ensembl

In order to confirm the evolutionary novelty of fish *TT*_*Rgr*_*EEN* genes we used an alternative ortholog search method, OMA (see Materials and Methods). Using this method, we detected 680 evolutionarily-novel genes in the sample of 870 fish genes expressed in tumors after regression. Of 409 Ensembl *TT*_*Rgr*_*EEN* genes, OMA confirmed 306 genes. OMA also confirmed all genes in Table 3 (see below).

**Fig. 2:**
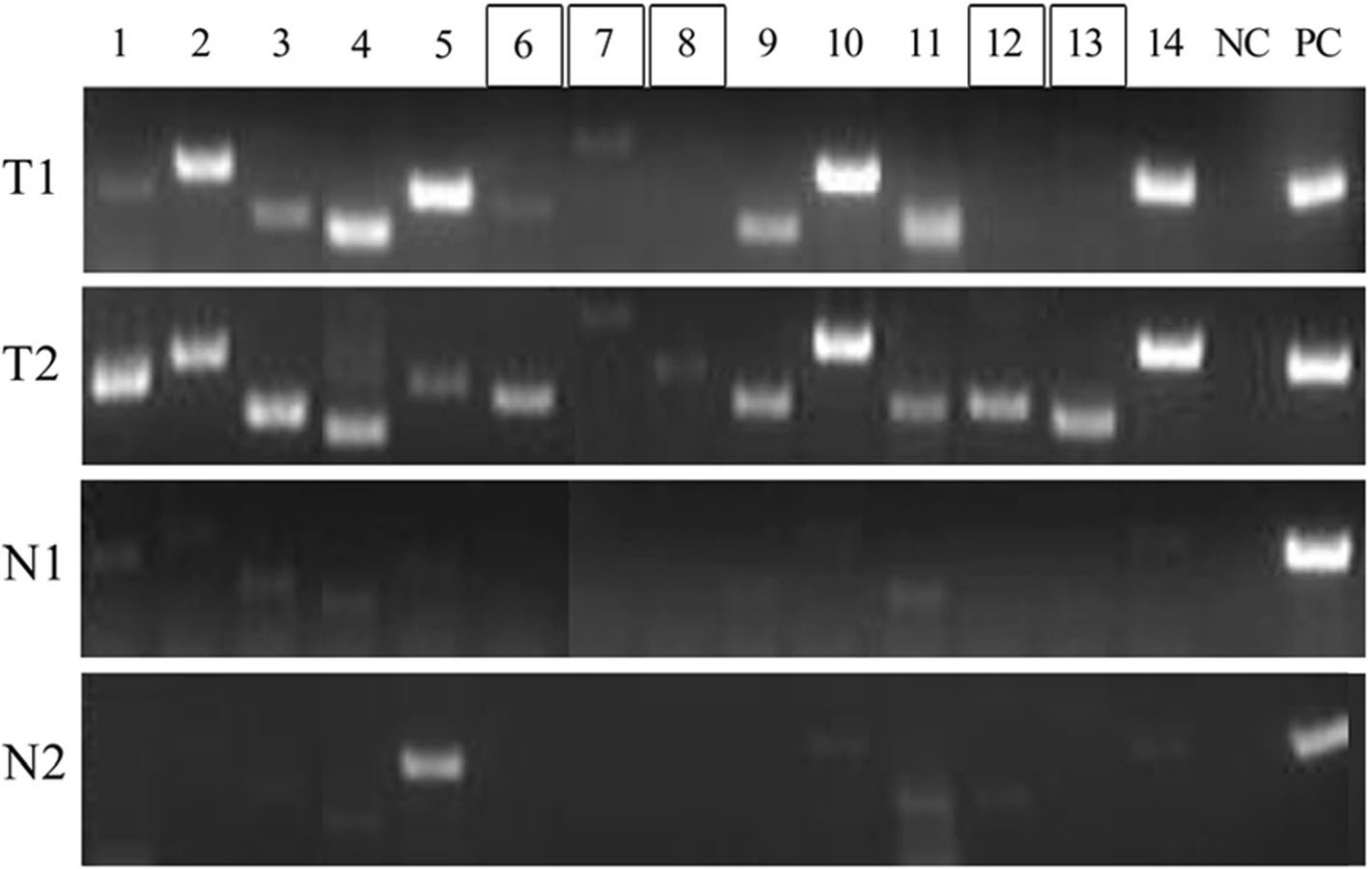
Confirmation of tumor-specific expression of fish *TT*_*Rgr*_*EEN* genes. Expression of zebrafish *TT*_*Rgr*_*EEN* genes in cDNA from zebrafish tumor and normal liver tissues. T1 - hepatocellular carcinoma, T2 - spermatocytic seminoma, N1, N2 - pooled normal liver. 1 – *camk4* – *Danio rerio* calcium/calmodulin-dependent protein kinase IV. 2 – *fus* – *Danio rerio* FUS RNA binding protein. 3 – *ssbp3a* – *Danio rerio* single stranded DNA binding protein 3a. 4 – *ripply1* – *Danio rerio* ripply transcriptional repressor 1. 5 – *tgfbrb2b* – *Danio rerio* transforming growth factor beta receptor 2b. 6 – *lepa* – *Danio rerio* leptin a. 7 – *sobpa* – *Danio rerio* sine oculis binding protein homolog (Drosophila) a. 8 – *ccdc40* – *Danio rerio* coiled-coil domain containing 40. 9 – *sema7a* – *Danio rerio* semaphorin 7A, transcript variant 2. 10 – *ephb3a* – *Danio rerio* eph receptor B3a. 11 – s*pry1* – *Danio rerio* sprouty homolog 1, antagonist of FGF signaling (Drosophila). 12 – *lmx1b* – *Danio rerio* LIM homeobox transcription factor 1, beta b. 13 – *nr2e1* – *Danio rerio* nuclear receptor subfamily 2, group E, member 1. 14 – *cacna1da*– *Danio rerio* calcium channel, voltage-dependent, L type, alpha 1D subunit, a. NC – no template control, PC – positive control – *gapdh – Danio rerio*

**Fig. 3:**
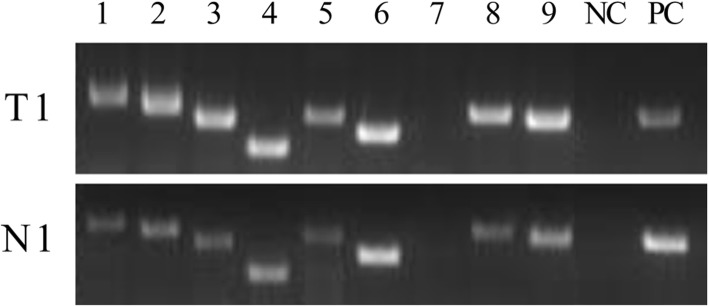
Expression of zebrafish *TT*_*Rgr*_*EEN* genes in cDNA from zebrafish tumor and normal liver tissues. T1- hepatocellular carcinoma, N1- pooled normal liver. 1 - *dazap1* - *Danio rerio* DAZ associated protein 1. 2 - *atxn1* - *Danio rerio* ataxin 1a. 3 - *wdtc1* - *Danio rerio* WD and tetratricopeptide repeats 1. 4 - *etnk2 - Danio rerio* ethanolamine kinase 2. 5 - *klf1* - *Danio rerio* Kruppel-like factor 1. 6 - *pbx4* - *Danio rerio* pre-B-cell leukemia transcription factor 4. 7 - *chrna4* - *Danio rerio* cholinergic receptor, nicotinic, alpha 4. 8 - *id2a* - *Danio rerio* inhibitor of DNA binding 2, dominant negative helix-loop-helix protein, a. 9 - *dhcr7* - *Danio rerio* 7-dehydrocholesterol reductase. NC – no template control, PC – positive control – *gapdh – Danio rerio*

To experimentally confirm the tumor-specific expression of some fish *TT*_*Rgr*_*EEN* genes identified as described above, we selected 12 genes from Table 3, 2 from Table 4 and 9 from the confirmed set of 306 *TT*_*Rgr*_*EEN* genes, and performed PCR with primers specific for these genes on cDNA from zebrafish normal and tumor tissues (Additional file 7). Histological data for fish tumor tissues are presented in Additional file 8*.* For 12 fish genes this analysis showed no or low expression in normal fish liver and increased expression in fish tumor tissues (Fig. 2), confirming the tumor-specific expression aspect of their *TSEEN* nature. For 7 other analyzed genes, overexpression in tumor tissues was detected (Fig. 3).

**Table 2:** Selected groups of functions determined by GO in different gene samples represented in Fig. 1

GO functions	Gene samples, studied with GO		
	343 Human orthologs of 296 fish TTRgrEEN genes	296 fish TTRgrEEN genes with human orthologs	113 fish TTRgrEEN genes without human orthologs
I Functions involved in developmental process			
Anatomical structure development			
	52	21	0
Developmental growth			
	181	25	4
Σ	233	46	4
II Functions involved in different aspects of transcription regulation			
Σ	111	78	15
III Functions involved in different signaling pathways			
Σ	117	86	21
IV Functions connected with immune system			
Σ	52	9	6
Σ Σ	513	219	46

The total number of functions determined by Gene Ontology for 296 fish *TT*_*Rgr*_*EEN* genes with human orthologs is 1813 and for 343 human orthologs is 6300. The 113 fish *TT*_*Rgr*_*EEN* genes with no human orthologs have 465 functions. The total number of functions for each gene was summed, skipping the repeated annotation in GO from different evidence codes. The total number of functions for each gene sample is the sum of all functions of all genes from this sample

In order to study the possible evolutionary appearance of new functions for the novel fish genes, we looked for human orthologs of the zebrafish Ensembl *TT*_*Rgr*_*EEN* genes, and found that the 296 fish *TT*_*Rgr*_*EEN* genes have 343 human orthologs, and that the remaining 113 fish Ensembl *TT*_*Rgr*_*EEN* genes have no human orthologs (Fig. 1). Hence, of 409 fish tumor-activated genes with no lamprey orthologs 296 (72.4%) have human orthologs (Fig. 1). Of the total 22,897 novel fish genes, as compared to lamprey, only 8230 (35.9%) are conserved in humans.

To estimate the possible functions of zebrafish *TT*_*Rgr*_*EEN* genes and their human orthologs, we used the Gene Ontology (GO) approach (Additional files 9, 10, 11 and 12 and Table 2). Gene samples studied by GO are those represented in Fig. 1. Additional file 9 contains gene ontology annotation with all evidence codes for all 870 tumor-activated fish genes with Ensemble IDs. Additional file 10 contains gene ontology annotation with all evidence codes for the 296 fish *TT*_*Rgr*_*EEN* genes with human orthologs. Additional file 11 contains gene ontology annotation with all evidence codes for 343 human orthologs of the 296 fish *TT*_*Rgr*_*EEN* genes. Gene ontology annotation for the 113 fish TTRgrEEN genes without human orthologs is in the Additional file 12.

GO functions are grouped in Table 2 as genes involved in developmental processes, genes involved in transcription, genes involved in different signaling pathways, and genes involved in the immune system. As we see from Table 2, fish *TT*_*Rgr*_*EEN* genes have fewer corresponding annotated functions than their human orthologs. The increase of the number of gene function annotations for human orthologs is especially evident for functions involved in developmental process and immune system, as compared to transcription regulation and signaling pathways (Additional file 13 contains full version of the Table 2). Some of 296 fish *TT*_*Rgr*_*EEN* genes have annotated functions of DNA binding, sequence specific DNA binding and regulation of DNA-templated transcription. Their human orthologs, in addition to the above mentioned functions, have annotated functions of anatomical structure development, anatomical structure involved in morphogenesis, and development of particular cell types and organs.

Statistical analysis of the GO enrichment analysis of human orthologs of fish *TT*_*Rgr*_*EEN* genes was made with the PANTHER13.1 functional clustering tool [45]. As a background we used all human genes. Results are presented in Additional file 14. We discovered enriched functional-related morphogenetic gene groups, e.g. in anatomical structure development (Fold enrichment: 19,360, raw *P*-value 1.93х10^− 7^) and system development (Fold enrichment: 21,916, raw P-value 3.02х10^− 7^) (Additional file 14).

Among 343 human orthologs of fish *TT*_*Rgr*_*EEN* genes, we found genes with functions that constitute progressive traits evolved on the way to humans, which do not exist in fish (Table 3), e.g., genes of lung development, mammary gland development, mammalian placenta development, ventricular septum development, etc.

**Table 3:** Selected human orthologs of fish *TT*_*Rgr*_*EEN* genes with functions that do not exist in fish

Name of ene (Fish gene/Human gene)	GO domain			Selected GO progressive functions not encountered in fish ([Fish gene]/[Human gene])
	Molecular function (Fish gene/Human gene)	Cellular component (Fish gene/Human gene)	Biological process (Fish gene/Human gene)	
Fish tgfbr2b/Human *TGFBR2*	10/18	4/12	9/84	[NO]/[bronchus development, bronchus morphogenesis, embryo implantation, in utero embryonic development, lung development, lung lobe morphogenesis, lung morphogenesis, mammary gland morphogenesis, ventricular septum development]
Fish lepa/Human *LEP*	2/4	3/3	15/106	[NO]/[placenta development
Fish sema7a/Human *SEMA7A*	1/3	0/4	1/16	[NO]/[olfactory lobe development
Fish klf1/Human *KLF1*	3/7	1/2	3/6	[NO]/[maternal process involved in female pregnancy
Fish ephb3a/Human *EPHB3*	7/9	3/8	5/25	[NO]/corpus callosum development
Fish dazap1/Human *DAZAP1*	2/6	0/6	0/6	[NO]/maternal placenta development
Fish spry1/Human *SPRY1*	0/1	1/6	6/16	[NO]/bud elongation involved in lung branching
Fish lmx1bb/Human *LMX1B*	3/7	1/1	13/9	[NO]/in utero embryonic development
Fish nr2e1/Human *NR2E1*	5/9	1/2	2/41	[NO]/cerebral cortex development, cerebral cortex neuron differentiation, dentate gyrus development, layer formation in cerebral cortex
Fish sobpa/Human *SOBP*	0/2	0/1	0/5	[NO]/cochlea development
Fish ccdc40/Human *CCDC40*	0/0	4/5	11/14	[NO]/lung development
Fish fosl1a/Human *FOSL1*	0/7	0/6	0/29	[NO]/placenta blood vessel development

OMA confirmed the evolutionary novelty of fish genes listed in Table 3 and added more human orthologs of fish *TT*_*Rgr*_*EEN* genes with functions not encountered in fish (Table 4).

**Table 4:** Additional human orthologs of fish *TT*_*Rgr*_*EEN* genes, according to OMA ortholog search algorithm, with functions that do not exist in fish

Name of gene (Fish gene/Human gene)	GO domain			Selected GO progressive functions not encountered in fish
	Molecular function (Fish gene/Human gene)	Cellular component (Fish gene/Human gene)	Biological process (Fish gene/Human gene)	([Fish gene]/[Human gene])
Fish atxn1l/Human *ATXN1L*	1 / 3	1 / 5	0 / 10	[NO] / lung alveolus development
Fish id2a/Human *ID2*	1 / 3	2 / 4	8 / 56	[NO]/epithelial cell differentiation involved in mammary gland alveolus development, mammary gland epithelial cell proliferation, mammary gland alveolus development, ventricular septum development
Fish ccr11.1/Human *CX3CR1*	3 / 4	2 / 7	4 / 17	[NO] / cerebral cortex cell migration
HFish cntnap2a/Human *CNTNAP2*	0 / 2	2 / 15	1 / 8	[NO] / cerebral cortex development
Fish mycn/Human *MYCN*	2 / 7	1 / 3	1 / 20	[NO] / lung development
Fish neflb/Human *NEFL*	1 / 10	2 / 10	1 / 29	[NO] / cerebral cortex development
Fish notch1b/Human *NOTCH1*	3 / 15	1 / 20	15 / 162	[NO] / lung development
Fish notch1b/Human *NOTCH2*	0 / 5	0 / 4	7 / 8	[NO] / embryo implantation
Fish notch1b/Human *NOTCH3*	2 / 7	2 / 11	4 / 40	[NO] / cerebral cortex development
Fish notch1b/Human *NOTCH4*	2 / 3	3 / 9	4 / 42	[NO]/trachea cartilage morphogenesis, lobar bronchus development, lung epithelium development, lung development, lung morphogenesis, chorio-allantoic fusion, embryonic placenta morphogenesis, mammary gland epithelium development
Fish notch1b/Human *NOTCH5*	7 / 30	2 / 8	6 / 81	[NO]/placenta development

To exclude the possibility of activation of the expression of 12 genes from Table 3 by mifepristone (which was used to induce tumorigenesis in our transgenic model), normal (non-transgenic) zebrafishes were treated with 5 υM mifepristone for 5 days, and their total RNA was studied in qPCR with specific primers. The results are presented in Additional file 16. From the presented data it is evident that mifepristone does not stimulate the expression of these genes in normal, nontransgenic fishes.

**Fig. 4:**
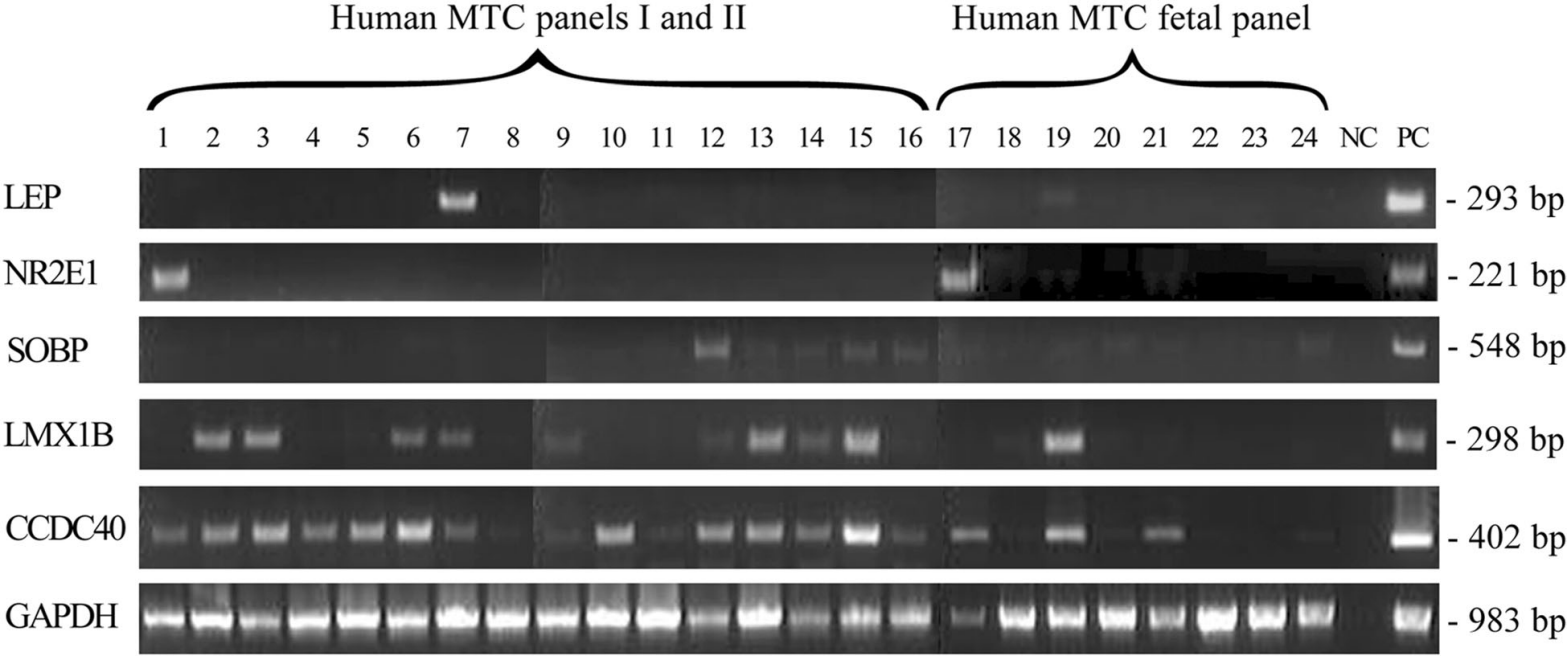
Expression of human orthologs of fish *TT*_*Rgr*_*EEN* genes from Table 3 in cDNA panels from human normal tissues. *LEP* – leptin*; NR2E1* – nuclear receptor subfamily 2 group E member 1*; SOBP* –sine oculis binding protein homolog*; LMX1B* – LIM homeobox transcription factor 1 beta*; CCDC40* – coiled-coil domain containing 40. Columns are Human MTC Panel I [1–8], Human MTC Panel II [9–16], Human Fetal MTC Panel [17–22, 28, 29]. 1 – brain, 2 – heart, 3 – kidney, 4 – liver, 5 – lung, 6 – pancreas, 7 – placenta, 8 – skeletal muscle, 9 – colon, 10 – ovary, 11 – peripheral blood leukocyte, 12 – prostate, 13 – small intestine, 14 – spleen, 15 – testis, 16 – thymus, 17 – fetal brain, 18 – fetal heart, 19 – fetal kidney, 20 – fetal liver, 21 – fetal lung, 22 – fetal skeletal muscle, 23 – fetal spleen, 24 – fetal thymus. NC – no template control. Lower pane: GAPDH control

**Fig. 5:**
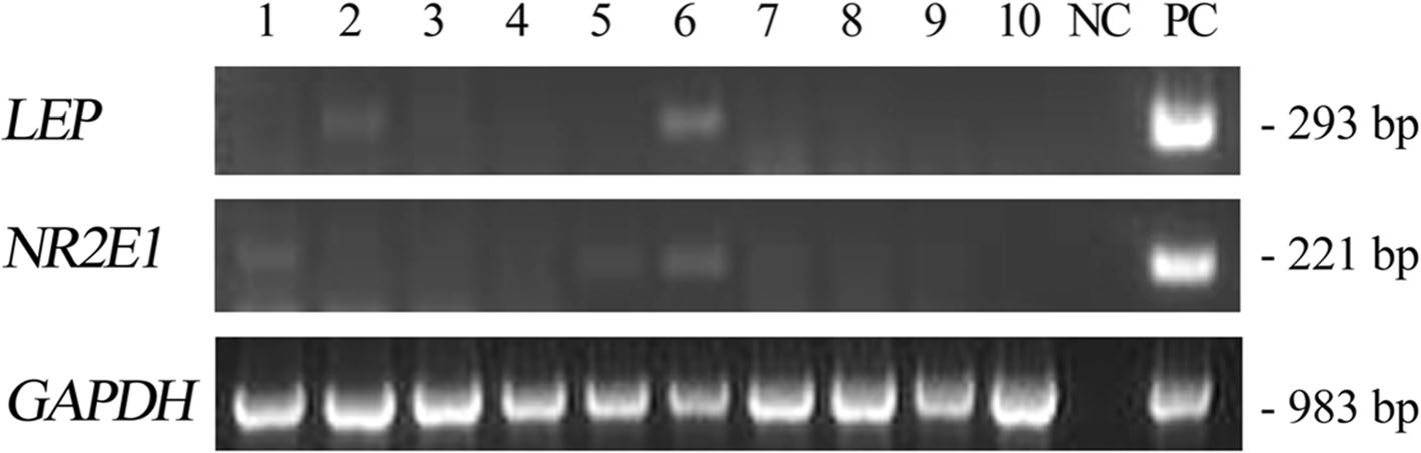
Expression of human orthologs of fish *TT*_*Rgr*_*EEN* genes in cDNA panels from human tumor tissues. LEP – leptin; NR2E1 – nuclear receptor subfamily 2 group E member 1. Tumor cDNA Panel: 1 – brain malignant meningioma moderately differentiated, 2 – breast invasive ductal carcinoma, 3 – colon adenocarcinoma, well differentiated, 4 – kidney renal cell carcinoma papillary, 5 – lung squamous cell carcinoma, well differentiated, 6 – ovary teratoma, 7 – pancreas adenocarcinoma, 8 – prostate adenocarcinoma, 9 – stomach adenocarcinoma, 10 – uterus leiomyoma. NC – no template control, PC – PCR with human DNA. Lower pane: GAPDH control

We experimentally verified the expression of several human genes from Table 3 (i.e. those which were selected both by Ensembl and OMA) in normal human tissues. We detected the expression of human gene *LEP*, the function of which is connected with the human placenta according to GO, in human placenta. Likewise, human gene *NR2E1*, the function of which is connected with brain according to GO, is expressed in brain and fetal brain tissue. *LMX1B* is expressed in placenta in accordance with GO (Fig. 4). On the contrary, *LEP* and *NR2E1* genes have little or no expression in human tumors (Fig. 5).

## Discussion

The hypothesis of the possible evolutionary role of tumors was recently published by A.P. Kozlov («Evolution by Tumor Neofunctionalization», [26]). According to this hypothesis, heritable tumors at earlier stages of progression, or heritable benign tumors may play an evolutionary role by providing extra cell masses that allow or promote the expression of evolutionarily-novel genes and can contribute to the origin of new cell types, tissues and organs. It is presumed that novel genes originate in the germline, not in tumor cells, that is why the new cell type is inherited in progeny generations. In this theory tumors are considered as search engines for expression of evolutionary novel and evolving genes, and of new combinations of genes [25, 26].

Tumors have features that could be used in evolution. Tumors are excessive cell masses which are functionally not necessary to the organism. Many unusual genes and gene combinations are activated in tumors. Tumors may differentiate with the loss of malignancy and have morphogenetic potential.

Many tumors never kill their hosts. Benign tumors are widespread in nature, and the available data suggest that tumors are represented throughout phylogenetic tree. Tumors may also be selected at earlier stages of progression for organismal functions. The list of tumors which could be used in evolution, discussed in [26], includes fetal, neonatal and infantile tumors; carcinomas in situ and pseudodiseases; tumors that spontaneously regress, and sustainable tumor masses. Examples of tumors that have played roles in evolution include eutherian placenta, the hoods of goldfishes, root nodules in legumes, symbiovilli of voles, macromelanophores of Xiphophorus fishes, head outgrowths in hardosaurs and prepupal horn primordia in beetles. These and other examples have been discussed in [26], and the list of such examples may be continued.

The widespread occurrence of tumors (discussed in [26] and in subsequent reviews [2, 3]); the hereditary nature of many tumors; the evidence that considerable proportion of tumors never kill their hosts; the features of tumors that could be used in evolution; and the presence of common features of tumors that are shared with embryonic development – all this evidence suggests that hereditary tumors could play positive role in evolution of host organisms, like mutational process did.

Tumors may be considered as atypical organs: they consist of parenchyma and stroma, they have vasculature and a hierarchy of cancer stem cells and more differentiated cells [17]. Hence, we have suggested that atypical tumor organs could evolve to normal organs in the course of progressive evolution [26].

The model of transgenic inducible fish hepatoma can be considered as an approximate model of an evolving organ, because the tumor scar after regression contains parenchyma cells different from normal liver parenchyma and stromal cells [34]. Thus we consider liver after tumor regression as a model of a «new» evolving organ. Such consideration is supported by a recent publication [31] that showed reversion of tumor hepatocytes to normal hepatocytes, although with somewhat different properties, during liver tumor regression in an oncogene transgenic zebrafish model. That is why we performed a study of expression of evolutionary novel genes in transgenic zebrafish tumors after their regression in experimental conditions.

RNA deep sequencing analysis demonstrated that at least 1502 genes not expressed in normal liver are expressed in liver cells after tumor regression. Annotations of 870 of these 1502 genes are found in the Ensembl database. The search for lamprey orthologs of these genes, by three different approaches, demonstrated that many of them may have no lamprey orthologs, implying that they are relatively evolutionarily-novel in fish (Table 1 and Fig. 1). Thus, based on Ensembl, there are 409 zebrafish tumor-activated genes without lamprey orthologs. These are tumor and tumor after regression expressed, evolutionarily novel (*TT*_*Rgr*_*EEN)* genes.

We understand that comparison only with the lamprey genome has its limitations, because lamprey may have lost some genes, which were present in other ancestral species. For example, *tapt1a* gene is lost in lamprey genome but present in other genomes (ascidian *Ciona savignyi, Ciona intestinalis* etc.) and may be considered as evolutionary conserved. However, after substraction this gene was in the list of 409 zebrafish genes without lamprey homologs and therefore could be classified as novel for fishes as compared to lamprey. This is an example of a gene with complex evolutionary history which may not have a single well defined age [11]. That is why we also used another method of estimation of gene orthology, OMA (Orthologous MAtrix). This method uses a reciprocal best hit approach and determines evolutionary distances instead of scores. It also considers the uncertainty of distance inference, includes many-to-many orthologous relations, and accounts the differential gene loss. Using OMA, we obtained a larger number of fish *TT*_*Rgr*_*EEN* genes (680) from the sample of 870 fish genes expressed in fish tumors after regression. This is probably because direct alignment, which determined the ortholog in the lamprey genome, was not confirmed by reverse alignment, which determined the reciprocal homology in the fish genome. That decreased the number of orthologs in lamprey genome and increased the number of *TT*_*Rgr*_*EEN* genes, even though OMA operated with several genomes, not only lamprey genome (Additional file 17). According to Ensembl Genes 90 Zebrafish genes (GrCz11), there are 35,117 genes in zebrafish genome. Straightforward comparison with the lamprey genome qualifies 23,308 genes as evolutionary-novel, using this as the only criterion. Such a big proportion of evolutionary-novel (compared to lamprey) genes may be explained by the whole genome duplications that occurred in fishes [35].

We found that 296 of 409 zebrafish *TT*_*Rgr*_*EEN* genes have 343 human orthologs. The other 113 fish genes have no human orthologs, i.e. either were lost during evolution or appeared after divergence of the ancestors of fishes and humans. The fact that the number of human orthologs is greater than that of the 296 zebrafish genes is due to origin of additional genes by gene duplication, as shown by the gene gain/loss trees in Ensembl [21]. More fish *TT*_*Rgr*_*EEN* genes (72.37%) are conserved in humans vs. all evolutionary-novel zebrafish genes (35.94%), *P*-value X2 test: 6.47х10^− 52^. This may indicate the importance of expression in tumors for selection and conservation of novel genes. This is in accordance with positive selection of many human tumor-related genes in primate lineage (reviewed in [25, 26]).

Gene Ontology analysis of the 296 fish *TT*_*Rgr*_*EEN* genes with human orthologs shows much fewer number of morphogenetic/developmental, immunological and other annotated functions of those genes in the fish as compared to corresponding human genes (total 1813 annotated fish functions vs. total 6300 annotated human functions, Table 2). The 296 fish *TT*_*Rgr*_*EEN* genes with human orthologs may represent proto-genes which acquire additional functions during the evolution of higher vertebrates. Proto-genes are considered as gene precursors which have not acquired functions yet [12]. Since the increase of the number of annotated gene functions in human orthologs is especially evident for functions involved in developmental process and immune system, as compared to transcription regulation and signaling pathways, we may conclude that evolution of fish proto-gene functions in higher vertebrates involved more organismic than molecular functions.

GO enrichment analysis of human orthologs of fish *TT*_*Rgr*_*EEN* genes also showed functional themes involved in development, i.e. anatomical structure development and system development (Additional file 14).

Protein kinase, DNA binding and transcriptional regulation functions are all highly represented among fish *TT*_*Rgr*_*EEN* genes (Table 2). It is known that protein kinase, DNA binding and transcriptional regulation domains are the most common domains encoded by cancer genes [8, 19]. Thus, the original functions of many fish proto-genes and of some of their human orthologs could be related to carcinogenesis. Human orthologs of fish *TT*_*Rgr*_*EEN* genes include oncogenes (12 as determined using COSMIC database and 6 using TSG database [52]) and tumor suppressor genes (35 found in TSG database and 2 in TAG database [13]).

The acquisition of new progressive functions by fish proto*-*genes in non-fish vertebrates is supported by finding that human orthologs of some of fish *TT*_*Rgr*_*EEN* genes are involved in development of traits which do not exist in fish, such as mammalian placental development, ovulation from ovarian follicle, lung development, mammary gland development, cerebral cortex development, and ventricular septum development (Tables 3 and 4). These additional functions may have been added to a smaller set of original functions of fish proto-genes in the course of evolution, and may be related to original functions of DNA binding, transcription regulation and protein kinase activity.

The fish *tgfbr2b* (transforming growth factor beta receptor 2b) gene illustrates the addition of progressive functions in higher vertebrates (Additional file 18). TGFß receptors are serine/threonine kinases that activate transcription factors. In metazoans, the transforming growth factor ß (TGFß) receptor family is the only class of receptors with intrinsic serine/ threonine kinase activity. The TGFß receptors bind to ligands such as TGFß, activin, bone morphogenetic proteins (BMPs), and nodal that regulate developmental cell fate and proliferation. In humans, there are ~ 12 distinct receptors in TGFß family, which can be functionally divided into two classes (type I and type II). All have a similar overall structure, with a single membrane-spanning domain and an intracellular serine/threonine kinase domain [32]. In fishes, tgfbr2b has protein kinase activity and few other molecular and cellular functions (Additional file 18). In humans, among many developmental and morphogenetic functions, this gene acquired functions in lung, mammary gland and ventricular septum development, not encountered in fishes (Table 3 and Additional file 18).

Fishes lack ventricular septation. It evolved independently in mammals and in birds and crocodilians [39]. The origin of the right ventricle of the vertebrate heart needed, besides duplication of Hand gene, the recruitment of a novel population of precursor cells instead of the simple expansion of pre-existing precursor cells [37]. There are two heart fields with progenitor cells that participate in building the mammalian heart [9]. The Tgfß – Smad signaling pathway specifies the anterior heart field which forms the right ventricle of the heart and outflow tract where specialized expression of Hand 2 takes place [49]. Interestingly, TGFß mediates tumor suppression in normal cells and facilitates cancer progression in malignant cells [46].

The other examples are nr2e1, mycn, fosl1a and dazap1 genes. In fishes, GO determines only molecular functions of DNA binding, DNA binding transcription factor activity, regulation of transcription (DNA tempated or from RNA polymerase II promoter), nucleic acid binding and RNA binding (Additional files 19, 20, 21, 22). In humans, NR2E1, MYCN, FOSL1 and DAZAP1 acquired additional functions connected with differentiation and development, including cerebral cortex development, lung development, placenta blood vessel development and maternal placenta development (Table 3 and Additional files 19, 20, 21, 22). MYCN and FOSL1 are known as cellular oncogenes [7, 47].

It was already mentioned above that domains involved in DNA binding, transcriptional regulation and protein kinase activity are among most common domains that are encoded by cancer genes [19]. The addition of novel progressive functions may balance the original oncogenic potential of fish TSEEN proto-genes. The similar situation – that some evolutionarily novel genes appear to promote tumorigenesis while evolving new advantageous functions – was described by other authors [51]. Evolution of new advantageous function may bring negative effects for survival of organisms, as discussed in recent selection, pleiotropy and compensation hypothesis (SPC) [38].

Tumor specificity of expression in *Danio rerio* was experimentally confirmed in vitro for lepa, nr2e1, lmx1b, sobpa and ccdc40 fish genes from Table 3. The expression of their human orthologs from Table 3 (LEP, NR2E1, LMX1B, SOBP and CCDC40) was experimentally confirmed in normal human organs, which have no analogs in fish. For LEP and NR2E1 little or no expression in human tumors is experimentally confirmed. Thus at least some human orthologs of fish TTRgrEEN genes acquire progressive functions, are expressed in normal human tissues and not expressed in human tumors.

According to the GO database, the majority of progressive traits of the genes we studied were inferred from their mutant phenotypes. As far as progressive traits listed in Tables 3 and 4 do not exist in fishes, we may be sure that they will never be discovered in fish TTRgrEEN genes. The acquisition of progressive functions which are not encountered in fish by human orthologs suggests that the functional difference between fish and human orthologs is not due to accuracy of annotation (incomplete knowledge of functions of fish genes provided by GO approach), but represents the real biological phenomenon.

As we have shown in our previous works [6, 15, 20, 27, 40, 44] human TSEEN genes are expressed in wide variety of tumors. In a similar way, the 296 fish *TT*_*Rgr*_*EEN* genes may be expressed not only in hepatomas, but in tumors of other localizations as well, that could lead to the origin of wide variety of morphogenetic gene functions. This is supported by our experimental data on the expression of the fish *TT*_*Rgr*_*EEN* genes (Fig. 2) and of their human orthologs in various normal human tissues (Fig. 4).

## Conclusions

Here, we propose that many genes that are involved in development of progressive traits in humans (such as placenta, mammary gland, lungs, ventricular septum, etc.) originated as proto-genes in fish and were expressed in fish tumors and in tumors after regression. This is exactly what the theory of evolution by tumor neofunctionalization predicted [25, 26].

Some of the human orthologs of fish *TT*_*Rgr*_*EEN* genes are involved in development of several progressive traits. Thus *TGFBR2* participates in lung development, mammary gland morphogenesis and ventricular septum development; *ID2* – in mammary gland and ventricular septum development; and *WNT7B* – in lung development, placenta morphogenesis and mammary gland development. Conversely, development of some progressive traits involves several of the human orthologs of fish *TT*_*Rgr*_*EEN* genes. For example, *ETNK2*, *FOSL1*, *LEP* and *DAZAP1* all participate in placenta development; *TGFBR2*, *SOX9* and *SPRY1*– in lung development; *TGFBR2*, *ID2* and *SOX9*– in mammary gland development, etc., Additional files 18, 19, 20, 21 and 22.

We suggest to call fish evolutionarily-novel genes that are expressed in fish tumors and that acquire morphogenetic, developmental and other important functions involved in evolution of progressive traits in higher orders of vertebrates with increased complexity, “*carcino-evo-devo”* genes, to stress their role in evolution of ontogenesis [26] and as analogy with carcinoembryonic antigens described earlier by Abelev and co-authors [1]. Our preliminary data obtained using newly available fish transcriptomes in public databases suggest that the *carcino-evo-devo* genes described in this paper include *TSEEN* genes (e.g. *lepa*), cancer/testis antigen genes (e.g. *ccr11.1*), carcino-embryonic genes (e.g. *ephb3a*) and genes expressed in normal fish tissues other than liver (e.g. *id2a, reck*).

*Carcino-evo-devo* genes were predicted by the hypothesis of evolutionary role of tumors [24–27]. That is why their discovery in this paper may be considered as support of the hypothesis of evolution by tumor neofunctionalization [25, 26].

## Supplementary information

**Additional file 1.** The List of normal liver transcripts.

**Additional file 2.** The List of carcinoma transcripts.

**Additional file 3.** The List of tumor after regression transcripts.

**Additional file 4.** The List of housekeeping genes with known expressionlevel in normal liver.

**Additional file 5.** The List of manually curated sample of 1502 genes, not expressed in normal liver, expressed in tumors and in tumors after regression.

**Additional file 6.** Ensembl data for 870 genes expressed in tumors and in liver after tumor regression.

**Additional file 7.** Primer sequences used for PCR.

**Additional file 8.** Histological data for tissues of spontaneous hepatocellular carcinoma and spermatocytic seminoma.

**Additional file 9.** GO annotation with all evidence codes for 870 fish genes from the sample.

**Additional file 10.** GO annotation with all evidence codes for 296 fish evolutionary novel genes with human orthologs.

**Additional file 11.** GO annotation with all evidence codes for 343 human orthologs of 296 fish evolutionary novel genes.

**Additional file 12.** GO annotation with all evidence codes for the 113 fishevolutionary novel genes without human orthologs.

**Additional file 13: Table S2.** Full version.

**Additional file 14.** GO enrichment functional clustering, using Panther algorithm.

**Additional file 15.** Primers for qPCR on set of genes selected to study the possibility of mifepristone influence on gene expression

**Additional file 16.** Results of the study of gene expression in the presence and absence of mifepristone, Figure.

**Additional file 17 **680 *TT*_*reg*_*EEN* genes detected by OMA.

**Additional file 18.** GO annotation of fish TSEEN tgfbr2b and it’s human ortholog TGFBR2.

**Additional file 19.** GO annotation of fish TSEEN dazap1 and it’s human ortholog DAZAP1.

**Additional file 20.** GO annotation of fish TSEEN nr2e1 and it’s human ortholog NR2E1.

**Additional file 21.** GO annotation of fish TSEEN mycn and it’s human ortholog MYCN

**Additional file 22.** GO annotation of fish TSEEN fosl1a and it’s human ortholog FOSL1.

**Additional file 23.** Source code of Original scripts “Alignment BLAST”, (BLAST database creation, Fasta slasher, OMA parameters).

## Data Availability

All data generated or analysed during this study are included in this published article (and its supplementary information files).

## References

[1] Abelev GI, Perova S, Khramkova NI, Postnikova Z, Irlin I (1963). Embryonal serum alpha-globulin and its synthesis by transplantable mouse hepatomas. Transplant Bull.

[2] Aktipis C. Athena, Boddy Amy M., Jansen Gunther, Hibner Urszula, Hochberg Michael E., Maley Carlo C., Wilkinson Gerald S. (2015). Cancer across the tree of life: cooperation and cheating in multicellularity. Philosophical Transactions of the Royal Society B: Biological Sciences.

[3] Albuquerque TA, do Val LD, Doherty A, de Magalhães JP (2018). From humans to hydra: patterns of cancer across the tree of life. Biol Rev.

[4] Altschul SF, Madden TL, Schäffer AA, Zhang J, Zhang Z, Miller W, Lipman DJ (1997). Gapped BLAST and PSI-BLAST: a new generation of protein database search programs. Nucl Acids Res.

[5] Ashburner Michael, Ball Catherine A., Blake Judith A., Botstein David, Butler Heather, Cherry J. Michael, Davis Allan P., Dolinski Kara, Dwight Selina S., Eppig Janan T., Harris Midori A., Hill David P., Issel-Tarver Laurie, Kasarskis Andrew, Lewis Suzanna, Matese John C., Richardson Joel E., Ringwald Martin, Rubin Gerald M., Sherlock Gavin (2000). Gene Ontology: tool for the unification of biology. Nature Genetics.

[6] Baranova AV, Lobashev AV, Ivanov DV, Krukovskaya LL, Yankovsky NK, Kozlov AP (2001). In silico screening for tumor-specific expressed sequences in human genome.

[7] Bell Emma, Chen Lindi, Liu Tao, Marshall Glenn M., Lunec John, Tweddle Deborah A. (2010). MYCN oncoprotein targets and their therapeutic potential. Cancer Letters.

[8] Blume-Jensen Peter, Hunter Tony (2001). Oncogenic kinase signalling. Nature.

[9] Buckingham Margaret, Meilhac Sigolène, Zaffran Stéphane (2005). Building the mammalian heart from two sources of myocardial cells. Nature Reviews Genetics.

[10] Camacho Christiam, Coulouris George, Avagyan Vahram, Ma Ning, Papadopoulos Jason, Bealer Kevin, Madden Thomas L (2009). BLAST+: architecture and applications. BMC Bioinformatics.

[11] Capra John A., Stolzer Maureen, Durand Dannie, Pollard Katherine S. (2013). How old is my gene?. Trends in Genetics.

[12] Carvunis Anne-Ruxandra, Rolland Thomas, Wapinski Ilan, Calderwood Michael A., Yildirim Muhammed A., Simonis Nicolas, Charloteaux Benoit, Hidalgo César A., Barbette Justin, Santhanam Balaji, Brar Gloria A., Weissman Jonathan S., Regev Aviv, Thierry-Mieg Nicolas, Cusick Michael E., Vidal Marc (2012). Proto-genes and de novo gene birth. Nature.

[13] Chen Jia-Shing, Hung Wei-Shiang, Chan Hsiang-Han, Tsai Shaw-Jenq, Sun H. Sunny (2012). In silico identification of oncogenic potential of fyn-related kinase in hepatocellular carcinoma. Bioinformatics.

[14] Cock P. J. A., Antao T., Chang J. T., Chapman B. A., Cox C. J., Dalke A., Friedberg I., Hamelryck T., Kauff F., Wilczynski B., de Hoon M. J. L. (2009). Biopython: freely available Python tools for computational molecular biology and bioinformatics. Bioinformatics.

[15] Dobrynin Pavel, Matyunina Ekaterina, Malov S. V., Kozlov A. P. (2013). The Novelty of Human Cancer/Testis Antigen Encoding Genes in Evolution. International Journal of Genomics.

[16] Edgar R, Domrachev M, Lash AE (2002). Gene expression omnibus: NCBI gene expression and hybridization array data repository. Nucleic Acids Res.

[17] Egeblad M, Nakasone ES, Werb Z (2010). Tumors as organs: complex tissues that interface with the entire organism. Dev Cell.

[18] Fernandez AA, Morris MR (2008). Mate choice for more melanin as a mechanism to maintain functional oncogene. Proc Natl Acad Sci U S A.

[19] Futreal P. Andrew, Coin Lachlan, Marshall Mhairi, Down Thomas, Hubbard Timothy, Wooster Richard, Rahman Nazneen, Stratton Michael R. (2004). A census of human cancer genes. Nature Reviews Cancer.

[20] Galachyants Y, Kozlov AP (2009). CDD as a tool for discovery of specifically-expressed transcripts. Russ J AIDS Cancer Public Health.

[21] Herrero Javier, Muffato Matthieu, Beal Kathryn, Fitzgerald Stephen, Gordon Leo, Pignatelli Miguel, Vilella Albert J., Searle Stephen M. J., Amode Ridwan, Brent Simon, Spooner William, Kulesha Eugene, Yates Andrew, Flicek Paul (2016). Ensembl comparative genomics resources. Database.

[22] Kozlov A.P. (1979). Evolution of living organisms as a multilevel process. Journal of Theoretical Biology.

[23] Kozlov AP, Presnov EV, Maresin VM, Zotin AI (1987). Theoretical and mathematical aspects of morphogenesis.

[24] Kozlov A.P. (1996). Gene competition and the possible evolutionary role of tumours. Medical Hypotheses.

[25] Kozlov A.P. (2010). The possible evolutionary role of tumors in the origin of new cell types. Medical Hypotheses.

[26] Kozlov AP (2014). Evolution by tumor Neofunctionalization.

[27] Kozlov AP (2016). Expression of evolutionarily novel genes in tumors. Infect Agents Cancer.

[28] Kozlov AP, Galachyants YP, Dukhovlinov IV, Samusik NA, Baranova AV, Polev DE, Krukovskaya LL. Evolutionarily new sequences expressed in tumors. Infect Agent Cancer. 2006. 10.1186/1750-9378-1-8. 10.1186/1750-9378-1-8 PMC1779766 17189608

[29] Kozlov AP, Zabezhinski MA, Popovich IG, Polev DE, Shilov ES, Muriashev BV (2012). Hyperplastic skin growth on the head of goldfish À comparative oncology aspects. Probl Oncol (VoprosiOncologii).

[30] Krukovskaja L.L., Baranova A., Tyezelova T., Polev D., Kozlov A.P. (2005). Experimental Study of Human Expressed Sequences Newly Identified in Silico as Tumor Specific. Tumor Biology.

[31] Li Yan, Agrawal Ira, Gong Zhiyuan (2019). Reversion of tumor hepatocytes to normal hepatocytes during liver tumor regression in an oncogene-expressing transgenic zebrafish model. Disease Models & Mechanisms.

[32] Lim W, Mayer B, Pawson T (2015). Cell signaling: principles and mechanisms.

[33] Mi Huaiyu, Muruganujan Anushya, Thomas Paul D. (2012). PANTHER in 2013: modeling the evolution of gene function, and other gene attributes, in the context of phylogenetic trees. Nucleic Acids Research.

[34] Nguyen A. T., Emelyanov A., Koh C. H. V., Spitsbergen J. M., Parinov S., Gong Z. (2011). An inducible krasV12 transgenic zebrafish model for liver tumorigenesis and chemical drug screening. Disease Models & Mechanisms.

[35] Ohno S (1970). Evolution by gene duplication.

[36] O'Leary Nuala A., Wright Mathew W., Brister J. Rodney, Ciufo Stacy, Haddad Diana, McVeigh Rich, Rajput Bhanu, Robbertse Barbara, Smith-White Brian, Ako-Adjei Danso, Astashyn Alexander, Badretdin Azat, Bao Yiming, Blinkova Olga, Brover Vyacheslav, Chetvernin Vyacheslav, Choi Jinna, Cox Eric, Ermolaeva Olga, Farrell Catherine M., Goldfarb Tamara, Gupta Tripti, Haft Daniel, Hatcher Eneida, Hlavina Wratko, Joardar Vinita S., Kodali Vamsi K., Li Wenjun, Maglott Donna, Masterson Patrick, McGarvey Kelly M., Murphy Michael R., O'Neill Kathleen, Pujar Shashikant, Rangwala Sanjida H., Rausch Daniel, Riddick Lillian D., Schoch Conrad, Shkeda Andrei, Storz Susan S., Sun Hanzhen, Thibaud-Nissen Francoise, Tolstoy Igor, Tully Raymond E., Vatsan Anjana R., Wallin Craig, Webb David, Wu Wendy, Landrum Melissa J., Kimchi Avi, Tatusova Tatiana, DiCuccio Michael, Kitts Paul, Murphy Terence D., Pruitt Kim D. (2015). Reference sequence (RefSeq) database at NCBI: current status, taxonomic expansion, and functional annotation. Nucleic Acids Research.

[37] Olson EN (2006). Gene regulatory networks in the evolution and development of the heart.

[38] Pavlicev Mihaela, Wagner Günter P. (2012). A model of developmental evolution: selection, pleiotropy and compensation. Trends in Ecology & Evolution.

[39] Poelmann Robert E., Groot Adriana C. Gittenberger-de, Vicente-Steijn Rebecca, Wisse Lambertus J., Bartelings Margot M., Everts Sonja, Hoppenbrouwers Tamara, Kruithof Boudewijn P. T., Jensen Bjarke, de Bruin Paul W., Hirasawa Tatsuya, Kuratani Shigeru, Vonk Freek, van de Put Jeanne M. M. S., de Bakker Merijn A., Richardson Michael K. (2014). Evolution and Development of Ventricular Septation in the Amniote Heart. PLoS ONE.

[40] Polev Dmitrii E., Karnaukhova Iuliia K., Krukovskaya Larisa L., Kozlov Andrei P. (2014). ELFN1-AS1: A Novel Primate Gene with Possible MicroRNA Function Expressed Predominantly in Human Tumors. BioMed Research International.

[41] Polev DE, Krukovskaya LL, Kozlov AP (2011). Locus Hs.633957 expression in human gastrointestinal tract and tumors. Vopr Onkol.

[42] Polev D. E., Nosova Yu. K., Krukovskaya L. L., Baranova A. V., Kozlov A. P. (2009). Expression of transcripts corresponding to cluster Hs.633957 in human healthy and tumor tissues. Molecular Biology.

[43] Roth AC, Gonnet GH, Dessimoz C. Algorithm of OMA for large-scale orthology inference. BMC Bioinformatics. 2008. 10.1186/1471-2105-9-518. 10.1186/1471-2105-9-518 PMC2639434 19055798

[44] Samusik Nikolay, Krukovskaya Larisa, Meln Irina, Shilov Evgeny, Kozlov Andrey P. (2013). PBOV1 Is a Human De Novo Gene with Tumor-Specific Expression That Is Associated with a Positive Clinical Outcome of Cancer. PLoS ONE.

[45] Thomas P. D. (2003). PANTHER: A Library of Protein Families and Subfamilies Indexed by Function. Genome Research.

[46] Tian Maozhen, Neil Jason R., Schiemann William P. (2011). Transforming growth factor-β and the hallmarks of cancer. Cellular Signalling.

[47] Vallejo Adrian, Valencia Karmele, Vicent Silvestre (2017). All for one and FOSL1 for all: FOSL1 at the crossroads of lung and pancreatic cancer driven by mutant KRAS. Molecular & Cellular Oncology.

[48] Vilella A. J., Severin J., Ureta-Vidal A., Heng L., Durbin R., Birney E. (2008). EnsemblCompara GeneTrees: Complete, duplication-aware phylogenetic trees in vertebrates. Genome Research.

[49] Von Both I, Silvestri C, Erdemir T, Lickert H, Walls JR, Henkelman RM, Rossant J, Harvey RP, Attisano L, Wrana JL (2004). Foxh1 is essential for development of the anterior heart field. Dev Cell.

[50] Westerfield M (2000). The Zebrafish Book. A guide for the laboratory use of Zebrafish (*Danio rerio*).

[51] Zhang Yong E, Long Manyuan (2014). New genes contribute to genetic and phenotypic novelties in human evolution. Current Opinion in Genetics & Development.

[52] Zhao Min, Sun Jingchun, Zhao Zhongming (2012). TSGene: a web resource for tumor suppressor genes. Nucleic Acids Research.

