# Correction to: Evolutionarily novel genes are expressed in transgenic fish tumors and their orthologs are involved in development of progressive traits in humans

**DOI:** 10.1186/s13027-020-0274-1

**Published:** 2020-01-27

**Authors:** E. A. Matyunina, A. V. Emelyanov, T. V. Kurbatova, A. A. Makashov, I. V. Mizgirev, A. P. Kozlov

**Affiliations:** 1 Research Institute of Ultra-Pure Biologicals, Ministry of Public Health of the Russian Federation, St.-Petersburg, Russia; 2 Peter the Great Saint-Petersburg Polytechnic University (SPbPU), St.-Petersburg, Russia; 3 The Biomedical Center (BMC), St.-Petersburg, Russia; 4 Institute for Research on Cancer and Aging (IRCAN), Nice, France; 5 Petrov Research Institute of Oncology, St.-Petersburg, Russia; 6 Vavilov Institute of General Genetics, Russian Academy of Sciences, Moscow, Russia

**Correction to: Infect Agents Cancer (2019) 14:46**

**https://doi.org/10.1186/s13027-019-0262-5**

The original publication of this article [1] contained 4 errors in column 1 of Table 4. In this correction article the errors and updated table are published.

**Table 4:** Additional human orthologs of fish *TT*_*Rgr*_*EEN* genes, according to OMA ortholog search algorithm, with functions that do not exist in fish

Name of gene (Fish gene / Human gene)	GO domain			Selected GO progressive functions not encountered in fish
	Molecular function (Fish gene / Human gene)	Cellular component (Fish gene / Human gene)	Biological process (Fish gene / Human gene)	([Fish gene] / [Human gene])
Fish atxn1l / Human *ATXN1L*	1 / 3	1 / 5	0 / 10	[NO] / lung alveolus development
Fish *id2a* / Human *ID2*	1 / 3	2 / 4	8 / 56	[NO] / epithelial cell differentiation involved in mammary gland alveolus development, mammary gland epithelial cell proliferation, mammary gland alveolus development, ventricular septum development
Fish *ccr11.1* / Human *CX3CR1*	3 / 4	2 / 7	4 / 17	[NO] / cerebral cortex cell migration
HFish *cntnap2a* /Human *CNTNAP2*	0 / 2	2 / 15	1 / 8	[NO] / cerebral cortex development
Fish *mycn* / Human *MYCN*	2 / 7	1 / 3	1 / 20	[NO] / lung development
Fish *neflb* / Human *NEFL*	1 / 10	2 / 10	1 / 29	[NO] / cerebral cortex development
Fish *notch1b* /Human *NOTCH1*	3 / 15	1 / 20	15 / 162	[NO] / lung development
Fish *reck* / Human *RECK*	0 / 5	0 / 4	7 / 8	[NO] / embryo implantation
Fish *srd5a1* / Human *SRD5A1*	2 / 7	2 / 11	4 / 40	[NO] / cerebral cortex development
Fish *wnt7bb* / Human *WNT7B*	2 / 3	3 / 9	4 / 42	[NO] / trachea cartilage morphogenesis, lobar bronchus development, lung epithelium development, lung development, lung morphogenesis, chorio-allantoic fusion, embryonic placenta morphogenesis, mammary gland epithelium development
Fish *pparg* / Human *PPARG*	7 / 30	2 / 8	6 / 81	[NO] / placenta development

**Errors in original publication:**

1. “Fish notch1b/Human NOTCH2” should be “Fish reck/Human RECK
2. “Fish notch1b/Human NOTCH3” should be “Fish srd5a1/Human SRD5A1
3. “Fish notch1b/Human NOTCH4” should be “Fish wnt7bb/ HumanWNT7B”
4. “Fish notch1b/Human NOTCH5” should be “Fish pparg/Human PPARG”
