# Supplementary material for: Evolutionarily novel genes are expressed in transgenic fish tumors and their orthologs are involved in development of progressive traits in humans
Source: Infect Agent Cancer. 2019 Dec 5;14:46. doi: 10.1186/s13027-019-0262-5 (PMC6896781; doi:10.1186/s13027-019-0262-5)
Supplement: Supplementary file 4 — Additional file 4. The List of housekeeping genes with known expressionlevel in normal liver. [file 13027_2019_262_MOESM4_ESM.doc]

**Table. The List of housekeeping genes with known expression level in normal liver.**

| Gene stable ID | Gene name | Gene description | RefSeq mRNA ID | GO term name | Gfold Readcount | FPKM (GFOLD) | Real time (Ct) |
| --- | --- | --- | --- | --- | --- | --- | --- |
| ENSDARG00000053136 | b2m | beta-2-microglobulin [Source:ZFIN;Acc:ZDB-GENE-980526-88] | NM_131163 | extracellular region | 1138 | 2.56033. | 23.8 ± 0.16 |
|  |  |  |  | immune response |  |  |  |
|  |  |  |  | immune system process |  |  |  |
|  |  |  |  | antigen processing and presentation of peptide antigen via MHC class I |  |  |  |
|  |  |  |  | MHC class I protein complex |  |  |  |
| ENSDARG00000020850 | eef1a1l2 | eukaryotic translation elongation factor 1 alpha 1, like 2 [Source:ZFIN;Acc:ZDB-GENE-050706-188] | NM_131263 | GTPase activity | 1753 | 2.8111 | 14.6 ± 0.12 |
|  |  |  |  | GTP binding |  |  |  |
|  |  |  |  | nucleotide binding |  |  |  |
|  |  |  |  | cytoplasm |  |  |  |
|  |  |  |  | intracellular |  |  |  |
|  |  |  |  | translation elongation factor activity |  |  |  |
|  |  |  |  | translational elongation |  |  |  |
|  |  |  |  | translation |  |  |  |
| ENSDARG00000004757 | ybx1 | Y box binding protein 1 [Source:ZFIN;Acc:ZDB-GENE-000629-3] | NM_131620 | nucleic acid binding | 1430 | 2.56033 |  |
|  |  |  |  | regulation of transcription, DNA-templated |  |  |  |
|  |  |  |  | DNA binding |  |  |  |
|  |  |  |  | RNA binding |  |  |  |
|  |  |  |  | eukaryotic initiation factor 4E binding |  |  |  |
|  |  |  |  | negative regulation of translational initiation |  |  |  |
|  |  |  |  | negative regulation of mRNA splicing, via spliceosome |  |  |  |
|  |  |  |  | negative regulation of mRNA processing |  |  |  |
|  |  |  |  | establishment of RNA localization |  |  |  |
|  |  |  |  | negative regulation of mRNA polyadenylation |  |  |  |
| ENSDARG00000091409 | zp2.6 | zona pellucida glycoprotein 2, tandem duplicate 6 [Source:ZFIN;Acc:ZDB-GENE-090306-2] | NM_131829 | extracellular region | 1244 | 2.8111. |  |
| ENSDARG00000037870 | actb2 | actin, beta 2 [Source:ZFIN;Acc:ZDB-GENE-000329-3] | NM_181601 | nucleotide binding | 1767 | 2.8111. |  |
|  |  |  |  | ATP binding |  |  |  |
|  |  |  |  | cytoplasm |  |  |  |
|  |  |  |  | cytoskeleton |  |  |  |
|  |  |  |  | plasma membrane |  |  |  |
|  |  |  |  | focal adhesion |  |  |  |
|  |  |  |  | dense body |  |  |  |
| ENSDARG00000103783 | cyyr1 | cysteine/tyrosine-rich 1 [Source:ZFIN;Acc:ZDB-GENE-040426-2356] | NM_212882 | membrane | 2928 | 2.8111. |  |
|  |  |  |  | integral component of membrane |  |  |  |
|  |  |  |  | molecular_function |  |  |  |
| ENSDARG00000039914 | gapdhs | glyceraldehyde-3-phosphate dehydrogenase, spermatogenic [Source:ZFIN;Acc:ZDB-GENE-020913-1] | NM_213094 | oxidation-reduction process | 1384 |  | 16.8 ± 0.12 |
|  |  |  |  | oxidoreductase activity |  |  |  |
|  |  |  |  | NAD binding |  |  |  |
|  |  |  |  | glycolytic process |  |  |  |
|  |  |  |  | NADP binding |  |  |  |
|  |  |  |  | glyceraldehyde-3-phosphate dehydrogenase (NAD+) (phosphorylating) activity |  |  |  |
|  |  |  |  | oxidoreductase activity, acting on the aldehyde or oxo group of donors, NAD or NADP as acceptor |  |  |  |
|  |  |  |  | glucose metabolic process |  |  |  |
| ENSDARG00000037746 | actb1 | actin, beta 1 [Source:ZFIN;Acc:ZDB-GENE-000329-1] | NM_131031 | nucleotide binding | 1683 |  | 15.1 ± 0.07 |
|  |  |  |  | ATP binding |  |  |  |
|  |  |  |  | cytoplasm |  |  |  |
|  |  |  |  | cytoskeleton |  |  |  |
|  |  |  |  | nucleus |  |  |  |
|  |  |  |  | cytoskeleton |  |  |  |
|  |  |  |  | plasma membrane |  |  |  |
|  |  |  |  | focal adhesion |  |  |  |
|  |  |  |  | dense body |  |  |  |
|  |  |  |  | actin cytoskeleton |  |  |  |
