# Supplementary material for: Evolutionarily novel genes are expressed in transgenic fish tumors and their orthologs are involved in development of progressive traits in humans
Source: Infect Agent Cancer. 2019 Dec 5;14:46. doi: 10.1186/s13027-019-0262-5 (PMC6896781; doi:10.1186/s13027-019-0262-5)
Supplement: Supplementary file 7 — Additional file 7. Primer sequences used for PCR. [file 13027_2019_262_MOESM7_ESM.doc]

Table. Primer sequences used for PCR.

| PCR primers targeting genes of zebrafish. | | | | |
| --- | --- | --- | --- | --- |
| № | Gene, NCBI Reference Sequence | Primer sequences | | Expected size of amplicon, bp |
| 1 | camk4 | for | 5’-GTTTGACAGGGTGGTGGAGAA- 3’ | 284 |
|  | [NM_001017607.1](https://www.ncbi.nlm.nih.gov/entrez/viewer.fcgi?db=nucleotide&id=62955182) | rev | 5’-CAGGACCATAGGCACATCCC- 3’ |  |
| 2 | fus | for | 5’-GGAGGACAGTCCGGTCAAAG- 3’ | 325 |
|  | [NM_201083.2](https://www.ncbi.nlm.nih.gov/entrez/viewer.fcgi?db=nucleotide&id=254553491) | rev | 5’-TATAGCCACCAGGGGCAGAT- 3’ |  |
| 3 | ssbp3a | for | 5’-CATAACCCTGGGTGAACCGC- 3’ | 230 |
|  | [NM_001130643.1](https://www.ncbi.nlm.nih.gov/entrez/viewer.fcgi?db=nucleotide&id=194578992) | rev | 5’-CAGGGGGTCCCTGAAAGAATCC- 3’ |  |
| 4 | ripply1 | for | 5’-CTATTCTCGACCAGCGCCAA- 3’ | 205 |
|  | [NM_001039108.1](https://www.ncbi.nlm.nih.gov/entrez/viewer.fcgi?db=nucleotide&id=84993743) | rev | 5’-TGGCCTAGAGTAAGGACAAGC- 3’ |  |
| 5 | tgfbr2 | for | 5’-TTAATGCGAGGGATACGGGC- 3’ | 278 |
|  | [NM_182855.3](https://www.ncbi.nlm.nih.gov/entrez/viewer.fcgi?db=nucleotide&id=402692664) | rev | 5’-ACTCTGCCTCCAGATAGCCA- 3’ |  |
| 6 | lepa | for | 5’-GGAACACATTGACGGGCAAAA- 3’ | 256 |
|  | [NM_001128576.1](https://www.ncbi.nlm.nih.gov/entrez/viewer.fcgi?db=nucleotide&id=190570285) | rev | 5’- GTCCAGCGCTTTCCCATTTG- 3’ |  |
| 7 | sobpa | for | 5’-AGATGAAGGTCCCGTCCAATC- 3’ | 407 |
|  | [NM_001098618.1](https://www.ncbi.nlm.nih.gov/entrez/viewer.fcgi?db=nucleotide&id=148762973) | rev | 5’-TGCTTTTATGAGAGGGGGCG- 3’ |  |
| 8 | ccdc40 | for | 5’-AGCAACAGAGATGCAAAGGGA- 3’ | 180 |
|  | [NM_001271813.1](https://www.ncbi.nlm.nih.gov/entrez/viewer.fcgi?db=nucleotide&id=425703049) | rev | 5’-CCTTCCATTTCCCAAAGTTTTCGT- 3’ |  |
| 9 | sema7a | for | 5’- ACCCAAGACCAGGAAAATGTGT- 3’ | 224 |
|  | [NM_001114885.2](https://www.ncbi.nlm.nih.gov/entrez/viewer.fcgi?db=nucleotide&id=1036551391) | rev | 5’-TCTATGTGTACCTGTCGCAAG- 3’ |  |
| 10 | ephb3a | for | 5’-GTCTTTATGATGACCCAGAGCG- 3’ | 339 |
|  | [NM_131097.1](https://www.ncbi.nlm.nih.gov/entrez/viewer.fcgi?db=nucleotide&id=40538749) | rev | 5’-TGCCAGCCTGTTTGAGTCG- 3’ |  |
| 11 | spry1 | for | 5’-CGTGTCTCTCAGGCTGTCA- 3’ | 228 |
|  | [NM_001122600.1](https://www.ncbi.nlm.nih.gov/entrez/viewer.fcgi?db=nucleotide&id=169790772) | rev | 5’-CTCTCGTGTTTGTGTGCGTG- 3’ |  |
| 12 | lmx1b | for | 5’-GTGCACGGTGTAGGAAAGGG- 3’ | 237 |
|  | [NM_001025167.2](https://www.ncbi.nlm.nih.gov/entrez/viewer.fcgi?db=nucleotide&id=309243110) | rev | 5’-CACTCCGTCCCGAGCATAA- 3’ |  |
| 13 | nr2e1 | for | 5’-TCAGCCCACACCGAAGTATC- 3’ | 218 |
|  | [NM_001003608.1](https://www.ncbi.nlm.nih.gov/entrez/viewer.fcgi?db=nucleotide&id=57525698) | rev | 5’-ACTGTGCAATACCCAGCACA- 3’ |  |
| 14 | cacna1da | for | 5’-ATGTATGCCAGGCAAGCTGT- 3’ | 343 |
|  | [NM_203484.1](https://www.ncbi.nlm.nih.gov/entrez/viewer.fcgi?db=nucleotide&id=45120099) | rev | 5’-GCAGGCGACTCTATGAGGAC- 3’ |  |
| 15 | [dazap1](https://www.ncbi.nlm.nih.gov/entrez/viewer.fcgi?db=nucleotide&id=291045423) | for | 5’-CTACAGGTCAACCGCTTGGG- 3’ | 353 |
|  | NM_001173504.1 | rev | 5’- CAGGTCCTGCCCATAACCATA- 3’ |  |
| 16 | [atxn1](https://www.ncbi.nlm.nih.gov/entrez/viewer.fcgi?db=nucleotide&id=113678035) | for | 5’-CCTGCCACAGCTATGGTTCA- 3’ | 324 |
|  | NM_001044826.1 | rev | 5’-TAGTACCTCCACACTGACCTGT- 3’ |  |
| 17 | [wdtc1](https://www.ncbi.nlm.nih.gov/entrez/viewer.fcgi?db=nucleotide&id=194578870) | for | 5’-CATGAAGCGCAAGTGGGATG- 3’ | 282 |
|  | NM_001130606.1 | rev | 5’-CGGATTGAGCTGTTCCCCTT- 3’ |  |
| 18 | [etnk2](https://www.ncbi.nlm.nih.gov/entrez/viewer.fcgi?db=nucleotide&id=77681589) | for | 5’-ACTTCCTCGGCTATGCAGTG- 3’ | 184 |
|  | NM_001034175.1 | rev | 5’-ACCCTATTCCCTCCCCTGAC- 3’ |  |
| 19 | klf1 | for | 5’-CAGTGGTCTTTCCTCCGCAT- 3’ | 304 |
|  | [NM_130936.1](https://www.ncbi.nlm.nih.gov/entrez/viewer.fcgi?db=nucleotide&id=18858936) | rev | 5’-TACGGCTTTTCACCTGTGTG- 3’ |  |
| 20 | pbx4 | for | 5’-CGACGCCAGACGTAAGAGAC- 3’ | 245 |
|  | [NM_131447.1](https://www.ncbi.nlm.nih.gov/entrez/viewer.fcgi?db=nucleotide&id=21915535) | rev | 5’-CGTCCACGGCTGTTTTAACG- 3’ |  |
| 21 | [chrna4](https://www.ncbi.nlm.nih.gov/entrez/viewer.fcgi?db=nucleotide&id=114326245) | for | 5’-GTGTTTTCCATGAGCCCAGC- 3’ | 316 |
|  | NM_001048063.1 | rev | 5’-CGTCCGCGTTGTTATAGAGC- 3’ |  |
| 22 | id2a | for | 5’-AGTAGCGCGAGTGTAACGAC- 3’ | 328 |
|  | [NM_201291.1](https://www.ncbi.nlm.nih.gov/entrez/viewer.fcgi?db=nucleotide&id=41152039) | rev | 5’-CAGGTGTCTGTAGTGAGAGGA- 3’ |  |
| 23 | dhcr7 | for | 5’-ACGTTTTGCAGGCCGTCTAT- 3’ | 311 |
|  | [NM_201330.1](https://www.ncbi.nlm.nih.gov/entrez/viewer.fcgi?db=nucleotide&id=41152206) | z | 5’-GAAGGTGGGCTTCTTACCCC- 3’ |  |
| 24 | gapdh | for | 5’-GTTGTAAGCAATGCCTCCTGC- 3’ | 324 |
|  | [NM_001115114.1](https://www.ncbi.nlm.nih.gov/entrez/viewer.fcgi?db=nucleotide&id=169403946) | rev | 5’-GGCAGGTTTCTCAAGACGGA- 3’ |  |
| PCR primers targeting human orthologs of fish *TSEEN* genes. | | | | |
| № | Gene, NCBI Reference Sequence | Primer sequences | | Expected size of amplicon, bp |
| 1 | LEP | for | 5’-ATGCCTCAATGTGACCAGGG- 3’ | 293 |
|  | [NM_000230.2](https://www.ncbi.nlm.nih.gov/entrez/viewer.fcgi?db=nucleotide&id=169790920) | rev | 5’-AGCTCAGCCAGACCCATCTA- 3’ |  |
| 2 | NR2E1 | for | 5’-ACGGGGTAATGAACTTCGGG- 3’ | 221 |
|  | [NM_003269.4](https://www.ncbi.nlm.nih.gov/entrez/viewer.fcgi?db=nucleotide&id=554790301) | rev | 5’-AGAGTGTGGGCAATCTCTGC- 3’ |  |
| 3 | SOBP | for | 5’-GACGGAAGATGGCTGACGAC- 3’ | 548 |
|  | [NM_018013.3](https://www.ncbi.nlm.nih.gov/entrez/viewer.fcgi?db=nucleotide&id=117938309) | rev | 5’-GCCATACCAGCCAAGGAGTT- 3’ |  |
| 4 | LMX1B | for | 5’-CCACCCTAAAGTCTGCCTGG- 3’ | 298 |
|  | [NM_001174147.1](https://www.ncbi.nlm.nih.gov/entrez/viewer.fcgi?db=nucleotide&id=292494914) | rev | 5’-CGGCAGAAGGACCAGAACAT- 3’ |  |
| 5 | CCDC40 | for | 5’-CCACCAGAGAAGGATGATGG- 3’ | 402 |
|  | [NM_017950.3](https://www.ncbi.nlm.nih.gov/UniGene/seq.cgi?ORG=Hs&SID=2293912) | rev | 5’-GCCTAAGACTCCGTGGGATG- 3’ |  |
| 6 | GAPDH | for | 5’-GAAGGTCGGAGTCAACGGATTTGGT-3’ | 982 |
|  | [NM_001289745.2](https://www.ncbi.nlm.nih.gov/entrez/viewer.fcgi?db=nucleotide&id=1276346089) | rev | 5’-CATGTGGGCCATGAGGTCCACCAC-3’ |  |
