# Supplementary material for: Evolutionarily novel genes are expressed in transgenic fish tumors and their orthologs are involved in development of progressive traits in humans
Source: Infect Agent Cancer. 2019 Dec 5;14:46. doi: 10.1186/s13027-019-0262-5 (PMC6896781; doi:10.1186/s13027-019-0262-5)
Supplement: Supplementary file 8 — Additional file 8. Histological data for tissues of spontaneous hepatocellular carcinoma and spermatocytic seminoma. [file 13027_2019_262_MOESM8_ESM.doc]

**Figure. Histology of zebrafish spontaneous tumors revealed in old fishes**


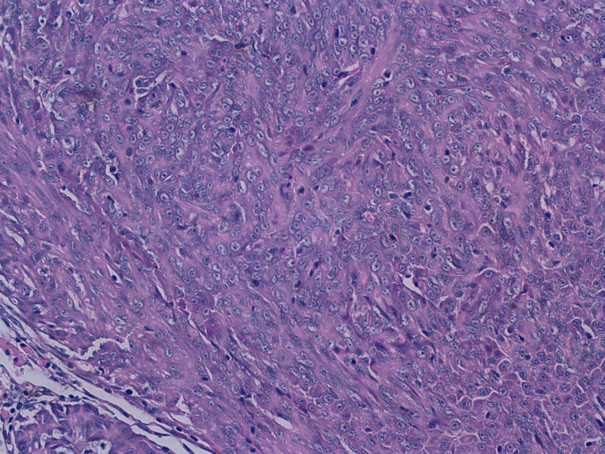

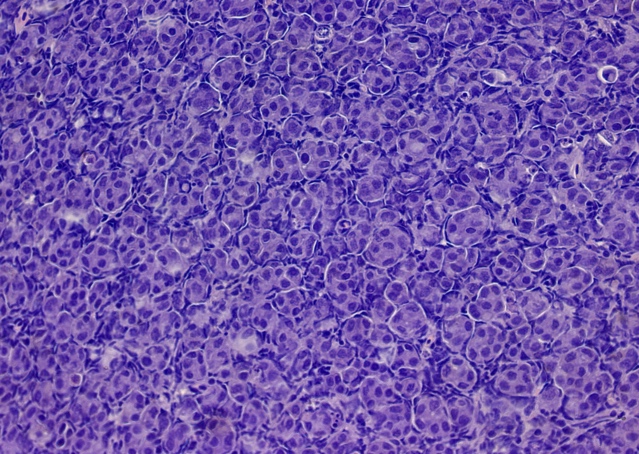


A

B

Paraffin sections 5mm thickness were stained by H&E.

1. A solid variant of hepatocellular carcinoma characterized by diffuse proliferation of neoplastic hepatocytes of variable size and shape with pleomorphic nuclei containing prominent nucleous;
2. High grade type spermatocytic seminoma characterized by the full loss of spermatocytic differentiation and abundant proliferation of poorly-differentiated spermatogonia-like cells.
