# Supplementary material for: Evolutionarily novel genes are expressed in transgenic fish tumors and their orthologs are involved in development of progressive traits in humans
Source: Infect Agent Cancer. 2019 Dec 5;14:46. doi: 10.1186/s13027-019-0262-5 (PMC6896781; doi:10.1186/s13027-019-0262-5)
Supplement: Supplementary file 13 — Additional file 13: Table S2. Full version. [file 13027_2019_262_MOESM13_ESM.doc]

Table – Table 2 full version

| **Gene samples, studied with GO**  **GO functions** | **343 Human orthologs of 296 fish TTRgrEEN genes** | **296 fish TTRgrEEN genes with human orthologs** | **113 fish TTRgrEEN genes without human orthologs** |
| --- | --- | --- | --- |
| Functions involved in developmental process | | | |
| Anatomical structure development | | | |
| brain development (adenohypophysis, amygdala, cerebellum, cerebral cortex, corpus callosum, dentate gyrus, habenula development, midbrain development, hindbrain development, forebrain development) | 23 | 4 | 0 |
| Heart development (atrial septum development, cardiac conduction system development, atrioventricular canal development, | 12 | 12 | 0 |
| head development | 4 | 0 | 0 |
| inner ear development | 4 | 3 | 0 |
| palate development | 2 | 1 | 0 |
| hair follicle development | 3 | 0 | 0 |
| endocrine pancreas development | 2 | 1 | 0 |
| developmental process involved in reproduction | 1 | 0 | 0 |
| paramesonephric duct development | 1 | 0 | 0 |
| Σ | 52 | 21 | 0 |
| Developmental growth | | | |
| organ growth (bone growth) | 1 | 0 | 0 |
| tissue development (connective tissue - cartilage - development; epithelium - endothelium development, otic vesicle formation; epidermis development) | 21 | 3 | 0 |
| multicellular organism development | 18 | 8 | 2 |
| nervous system development (central nervous system - pituitary gland, telencephalon, olfactory bulb, corpus callosum, substantia nigra development; autonomic nervous system - sympathetic ganglion, spinal cord development; neurogenesis - neuron development ) | 42 | 6 | 0 |
| circulatory system development (blood vessel, mitral valve, ventricular septum development) | 9 | 1 | 1 |
| skeletal system development | 11 | 1 | 1 |
| immune system development (spleen development) | 1 | 0 | 0 |
| urogenital system development (renal system, metanephric tubule, ureter development; nephric duct morphogenesis) | 29 | 0 | 0 |
| reproductive system development (cervix, oviduct development) | 2 | 0 | 0 |
| digestive system development (digestive tract development) | 2 | 0 | 0 |
| hepaticobiliary system development (intrahepatic bile duct development) | 3 | 2 | 0 |
| exocrine system development (lacrimal gland development) | 3 | 0 | 0 |
| respiratory system development (lung development, lung smooth muscle development) | 11 | 0 | 0 |
| male sex differentiation (male gonad development) | 4 | 1 | 0 |
| female sex differentiation (vagina development) | 1 | 0 | 0 |
| appendage development (limb development) | 1 | 0 | 0 |
| embryo development (embryonic organ development, embryo development ending in birth or egg hatching, somite rostral/caudal axis specification, neural tube development) | 5 | 0 | 0 |
| post-embryonic development (eye development, camera-type eye development, neural crest cell differentiation, mammary gland development, thymus development, placenta development, positive regulation of adipose tissue development) | 15 | 3 | 0 |
| cellular component organization or biogenesis. (negative regulation of neuron projection development) | 2 | 0 | 0 |
| Σ | 181 | 25 | 4 |
| Σ | 233 | 46 | 4 |
| Functions involved in different aspects of transcription regulation | | |  |
| transcription, DNA-templated | 42 | 20 | 7 |
| DNA binding | 27 | 36 | 5 |
| negative regulation of transcription from RNA polymerase II promoter | 11 | 1 | 0 |
| sequence-specific DNA binding | 11 | 7 | 3 |
| Transcription factor complex | 1 | 0 | 0 |
| transcription factor activity, sequence-specific DNA binding | 16 | 9 | 0 |
| RNA polymerase II transcription factor activity, sequence-specific DNA binding | 3 | 5 | 0 |
| Σ | 111 | 78 | 15 |
| Functions involved in different signaling pathways | | |  |
| apoptotic process | 39 | 9 | 4 |
| protein serine/threonine kinase activity | 8 | 12 | 2 |
| protein tyrosine kinase activity | 2 | 1 | 2 |
| calmodulin-dependent protein kinase activity | 1 | 1 | 0 |
| calcium-dependent protein serine/threonine kinase activity | 1 | 1 | 0 |
| kinase activity | 18 | 21 | 2 |
| protein kinase activity | 22 | 19 | 5 |
| G-protein coupled receptor activity | 12 | 12 | 6 |
| neurotrophin TRK receptor signaling pathway | 0 | 10 | 0 |
| intrinsic apoptotic signaling pathway | 5 | 0 | 0 |
| epidermal growth factor receptor signaling pathway | 1 | 0 | 0 |
| fibroblast growth factor receptor signaling pathway | 3 | 0 | 0 |
| rhodopsin mediated signaling pathway | 4 | 0 | 0 |
| vascular endothelial growth factor receptor signaling pathway | 1 | 0 | 0 |
| Σ | 117 | 86 | 21 |
| Functions connected with immune system | | | |
| innate immune response | 10 | 0 | 0 |
| positive regulation of innate immune response | 0 | 0 | 0 |
| innate immune response in mucosa | 0 | 1 | 0 |
| adaptive immune response | 0 | 0 | 0 |
| B cell activation involved in immune response, mature B cell differentiation involved in immune response | 1 | 0 | 0 |
| leukocyte activation involved in immune response | 1 | 0 | 0 |
| immune system process | 14 | 2 | 0 |
| immune response to tumor cell | 1 | 0 | 0 |
| negative regulation of immune system process | 1 | 0 | 0 |
| positive regulation of natural killer cell mediated immunity | 1 | 0 | 0 |
| regulation of T cell cytokine production | 1 | 2 | 0 |
| negative regulation of T cell cytokine production | 1 | 1 | 0 |
| immune response | 11 | 1 | 3 |
| complement activation | 2 | 1 | 0 |
| regulation of immune response | 3 | 0 | 0 |
| immunological synapse formation | 1 | 0 | 0 |
| immunological synapse | 1 | 0 | 0 |
| positive regulation of cytokine production | 1 | 1 | 0 |
| positive regulation of leukocyte mediated cytotoxicity | 1 | 0 | 0 |
| T cell mediated cytotoxicity | 1 | 0 | 3 |
| Σ | 52 | 9 | 6 |
| ΣΣ | 513 | 219 | 46 |
