# Supplementary material for: Evolutionarily novel genes are expressed in transgenic fish tumors and their orthologs are involved in development of progressive traits in humans
Source: Infect Agent Cancer. 2019 Dec 5;14:46. doi: 10.1186/s13027-019-0262-5 (PMC6896781; doi:10.1186/s13027-019-0262-5)
Supplement: Supplementary file 14 — Additional file 14. GO enrichment functional clustering, using Panther algorithm. [file 13027_2019_262_MOESM14_ESM.doc]

Table – GO enrichment functional clustering, using Panther algorithm.

| Analyzed List: | upload_1 (Homo sapiens) | | | | | | |
| --- | --- | --- | --- | --- | --- | --- | --- |
| Reference List: | Homo sapiens (all genes in database) | | | | | | |
| Test Type: | FISHER |  |  |  |  |  |  |
| GO biological process complete | Homo sapiens - REFLIST (21042) | upload_1 (337) | upload_1 (expected) | upload_1 (over/under) | upload_1 (fold Enrichment) | upload_1 (raw P-value) | upload_1 (FDR) |
| ethanol oxidation (GO:0006069) | 12 | 7 | .19 | + | 36.42 | 9.73E-09 | 1.51E-04 |
| ethanol metabolic process (GO:0006067) | 21 | 7 | .34 | + | 20.81 | 2.02E-07 | 4.48E-04 |
| cornification (GO:0070268) | 113 | 13 | 29.587 | + | 43.282 | 1.02E-07 | 5.25E-04 |
| visual perception (GO:0007601) | 207 | 17 | 11.749 | + | 41.395 | 1.09E-07 | 4.22E-04 |
| sensory perception of light stimulus (GO:0050953) | 210 | 17 | 13.210 | + | 43.225 | 1.32E-07 | 4.09E-04 |
| system development (GO:0048731) | 4173 | 107 | 66.83 | + | 21.916 | 3.02E-07 | 5.86E-04 |
| multicellular organism development (GO:0007275) | 4769 | 120 | 76.38 | + | 20.821 | 9.34E-08 | 7.24E-04 |
| anatomical structure development (GO:0048856) | 5137 | 126 | 82.27 | + | 19.360 | 1.93E-07 | 5.00E-04 |
| developmental process (GO:0032502) | 5497 | 128 | 88.04 | + | 16.438 | 2.52E-06 | 3.91E-03 |
| multicellular organismal process (GO:0032501) | 6688 | 151 | 107.11 | + | 14.977 | 8.80E-07 | 1.52E-03 |
