# Supplementary material for: Evolutionarily novel genes are expressed in transgenic fish tumors and their orthologs are involved in development of progressive traits in humans
Source: Infect Agent Cancer. 2019 Dec 5;14:46. doi: 10.1186/s13027-019-0262-5 (PMC6896781; doi:10.1186/s13027-019-0262-5)
Supplement: Supplementary file 16 — Additional file 16. Results of the study of gene expression in the presence and absence of mifepristone, Figure. [file 13027_2019_262_MOESM16_ESM.doc]

**Results of the study of gene expression in the presence and absence of mifepristone.**


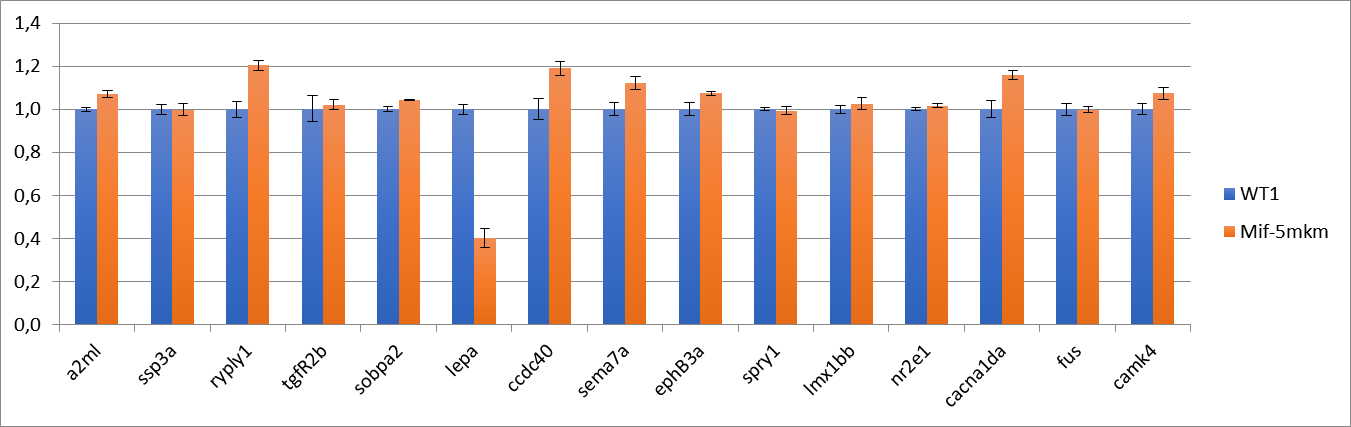


PCR results are described by fold change of the expression under mifepristone (y axis).
