# Supplementary material for: Evolutionarily novel genes are expressed in transgenic fish tumors and their orthologs are involved in development of progressive traits in humans
Source: Infect Agent Cancer. 2019 Dec 5;14:46. doi: 10.1186/s13027-019-0262-5 (PMC6896781; doi:10.1186/s13027-019-0262-5)
Supplement: Supplementary file 18 — Additional file 18. GO annotation of fish TSEEN tgfbr2b and it’s human ortholog TGFBR2. [file 13027_2019_262_MOESM18_ESM.doc]

Table – GO annotation of fish TSEEN tgfbr2b and its human ortholog TGFBR2

| Danio rerio |  | Human |  |
| --- | --- | --- | --- |
| Gene stable ID | Gene name | Gene stable ID | Gene name |
| ENSDARG00000034541 | tgfbr2b | ENSG00000163513 | TGFBR2 |
|  |  |  |  |
| GO term name | GO domain | GO term name | GO domain |
| ATP binding | molecular_function | ATP binding | molecular_function |
| kinase activity | molecular_function | glycosaminoglycan binding | molecular_function |
| metal ion binding | molecular_function | kinase activity | molecular_function |
| nucleotide binding | molecular_function | metal ion binding | molecular_function |
| protein kinase activity | molecular_function | mitogen-activated protein kinase kinase kinase binding | molecular_function |
| protein serine/threonine kinase activity | molecular_function | nucleotide binding | molecular_function |
| receptor activity | molecular_function | protein binding | molecular_function |
| signal transducer, downstream of receptor, with serine/threonine kinase activity | molecular_function | protein kinase activity | molecular_function |
| transferase activity | molecular_function | protein serine/threonine kinase activity | molecular_function |
| transforming growth factor beta receptor activity, type II | molecular_function | receptor activity | molecular_function |
| integral component of membrane | cellular_component | signal transducer, downstream of receptor, with serine/threonine kinase activity | molecular_function |
| membrane | cellular_component | SMAD binding | molecular_function |
| plasma membrane | cellular_component | transferase activity | molecular_function |
| receptor complex | cellular_component | transforming growth factor beta binding | molecular_function |
| apoptotic process | biological_process | transforming growth factor beta receptor activity, type II | molecular_function |
| cell differentiation | biological_process | transforming growth factor beta-activated receptor activity | molecular_function |
| hematopoietic stem cell differentiation | biological_process | transmembrane receptor protein serine/threonine kinase activity | molecular_function |
| phosphorylation | biological_process | type I transforming growth factor beta receptor binding | molecular_function |
| protein phosphorylation | biological_process | caveola | cellular_component |
| regulation of growth | biological_process | cell surface | cellular_component |
| signal transduction by protein phosphorylation | biological_process | cytoplasm | cellular_component |
| transforming growth factor beta receptor signaling pathway | biological_process | cytosol | cellular_component |
| transmembrane receptor protein serine/threonine kinase signaling pathway | biological_process | external side of plasma membrane | cellular_component |
|  |  | integral component of membrane | cellular_component |
|  |  | integral component of plasma membrane | cellular_component |
|  |  | membrane | cellular_component |
|  |  | membrane raft | cellular_component |
|  |  | plasma membrane | cellular_component |
|  |  | receptor complex | cellular_component |
|  |  | transforming growth factor beta receptor complex | cellular_component |
|  |  | activation of protein kinase activity | biological_process |
|  |  | aging | biological_process |
|  |  | animal organ morphogenesis | biological_process |
|  |  | animal organ regeneration | biological_process |
|  |  | apoptotic process | biological_process |
|  |  | atrioventricular valve morphogenesis | biological_process |
|  |  | blood vessel development | biological_process |
|  |  | brain development | biological_process |
|  |  | branching involved in blood vessel morphogenesis | biological_process |
|  |  | bronchus development | biological_process |
|  |  | bronchus morphogenesis | biological_process |
|  |  | cardiac left ventricle morphogenesis | biological_process |
|  |  | cartilage development | biological_process |
|  |  | cell differentiation | biological_process |
|  |  | common-partner SMAD protein phosphorylation | biological_process |
|  |  | digestive tract development | biological_process |
|  |  | embryo implantation | biological_process |
|  |  | embryonic cranial skeleton morphogenesis | biological_process |
|  |  | embryonic hemopoiesis | biological_process |
|  |  | endocardial cushion fusion | biological_process |
|  |  | gastrulation | biological_process |
|  |  | growth plate cartilage development | biological_process |
|  |  | heart development | biological_process |
|  |  | heart looping | biological_process |
|  |  | in utero embryonic development | biological_process |
|  |  | inferior endocardial cushion morphogenesis | biological_process |
|  |  | lens development in camera-type eye | biological_process |
|  |  | lens fiber cell apoptotic process | biological_process |
|  |  | lung development | biological_process |
|  |  | lung lobe morphogenesis | biological_process |
|  |  | lung morphogenesis | biological_process |
|  |  | mammary gland morphogenesis | biological_process |
|  |  | membranous septum morphogenesis | biological_process |
|  |  | miRNA transport | biological_process |
|  |  | myeloid dendritic cell differentiation | biological_process |
|  |  | negative regulation of cardiac muscle cell proliferation | biological_process |
|  |  | negative regulation of cell proliferation | biological_process |
|  |  | negative regulation of transforming growth factor beta receptor signaling pathway | biological_process |
|  |  | Notch signaling pathway | biological_process |
|  |  | outflow tract morphogenesis | biological_process |
|  |  | outflow tract septum morphogenesis | biological_process |
|  |  | palate development | biological_process |
|  |  | pathway-restricted SMAD protein phosphorylation | biological_process |
|  |  | peptidyl-serine phosphorylation | biological_process |
|  |  | peptidyl-threonine phosphorylation | biological_process |
|  |  | phosphorylation | biological_process |
|  |  | positive regulation of angiogenesis | biological_process |
|  |  | positive regulation of B cell tolerance induction | biological_process |
|  |  | positive regulation of CD4-positive, alpha-beta T cell proliferation | biological_process |
|  |  | positive regulation of cell proliferation | biological_process |
|  |  | positive regulation of epithelial cell migration | biological_process |
|  |  | positive regulation of epithelial to mesenchymal transition | biological_process |
|  |  | positive regulation of epithelial to mesenchymal transition involved in endocardial cushion formation | biological_process |
|  |  | positive regulation of mesenchymal cell proliferation | biological_process |
|  |  | positive regulation of NK T cell differentiation | biological_process |
|  |  | positive regulation of reactive oxygen species metabolic process | biological_process |
|  |  | positive regulation of skeletal muscle tissue regeneration | biological_process |
|  |  | positive regulation of smooth muscle cell proliferation | biological_process |
|  |  | positive regulation of T cell tolerance induction | biological_process |
|  |  | positive regulation of tolerance induction to self antigen | biological_process |
|  |  | protein phosphorylation | biological_process |
|  |  | receptor-mediated endocytosis | biological_process |
|  |  | regulation of cell proliferation | biological_process |
|  |  | regulation of gene expression | biological_process |
|  |  | regulation of growth | biological_process |
|  |  | response to cholesterol | biological_process |
|  |  | response to drug | biological_process |
|  |  | response to estrogen | biological_process |
|  |  | response to glucose | biological_process |
|  |  | response to hypoxia | biological_process |
|  |  | response to mechanical stimulus | biological_process |
|  |  | response to nutrient | biological_process |
|  |  | response to organic cyclic compound | biological_process |
|  |  | response to organic substance | biological_process |
|  |  | response to steroid hormone | biological_process |
|  |  | signal transduction by protein phosphorylation | biological_process |
|  |  | smoothened signaling pathway | biological_process |
|  |  | trachea formation | biological_process |
|  |  | trachea morphogenesis | biological_process |
|  |  | transforming growth factor beta receptor signaling pathway | biological_process |
|  |  | transmembrane receptor protein serine/threonine kinase signaling pathway | biological_process |
|  |  | tricuspid valve morphogenesis | biological_process |
|  |  | vasculogenesis | biological_process |
|  |  | ventricular septum morphogenesis | biological_process |
|  |  | wound healing | biological_process |
