# Supplementary material for: Evolutionarily novel genes are expressed in transgenic fish tumors and their orthologs are involved in development of progressive traits in humans
Source: Infect Agent Cancer. 2019 Dec 5;14:46. doi: 10.1186/s13027-019-0262-5 (PMC6896781; doi:10.1186/s13027-019-0262-5)
Supplement: Supplementary file 19 — Additional file 19. GO annotation of fish TSEEN dazap1 and it’s human ortholog DAZAP1. [file 13027_2019_262_MOESM19_ESM.doc]

**Table – GO annotation of fish TSEEN dazap1 and it’s human ortholog DAZAP1**

| DAZ associated protein 1 (dazap1) |  | DAZ associated protein 1 (DAZAP1) |  |
| --- | --- | --- | --- |
| Danio rerio |  | Human |  |
| Gene stable ID | Gene name | Gene stable ID | Gene name |
| ENSDARG00000070846 | dazap1 | ENSG00000071626 | DAZAP1 |
|  |  |  |  |
| GO term name | GO domain | GO term name | GO domain |
| nucleic acid binding | molecular_function | nucleic acid binding | molecular_function |
| RNA binding | molecular_function | poly(G) binding | molecular_function |
|  |  | poly(U) RNA binding | molecular_function |
|  |  | protein binding | molecular_function |
|  |  | RNA binding | molecular_function |
|  |  | RNA stem-loop binding | molecular_function |
|  |  | cytoplasm | cellular_component |
|  |  | cytosol | cellular_component |
|  |  | macromolecular complex | cellular_component |
|  |  | nucleoplasm | cellular_component |
|  |  | nucleus | cellular_component |
|  |  | ribonucleoprotein complex | cellular_component |
|  |  | cell differentiation | biological_process |
|  |  | cell proliferation | biological_process |
|  |  | maternal placenta development | biological_process |
|  |  | multicellular organism development | biological_process |
|  |  | positive regulation of mRNA splicing, via spliceosome | biological_process |
|  |  | spermatogenesis | biological_process |
