# Supplementary material for: Evolutionarily novel genes are expressed in transgenic fish tumors and their orthologs are involved in development of progressive traits in humans
Source: Infect Agent Cancer. 2019 Dec 5;14:46. doi: 10.1186/s13027-019-0262-5 (PMC6896781; doi:10.1186/s13027-019-0262-5)
Supplement: Supplementary file 20 — Additional file 20. GO annotation of fish TSEEN nr2e1 and it’s human ortholog NR2E1. [file 13027_2019_262_MOESM20_ESM.doc]

**Table – GO annotation of fish TSEEN nr2e1 and it’s human ortholog NR2E1**

| *Danio rerio* | | *Homo sapiens* | |
| --- | --- | --- | --- |
| *nr2e1* (ENSDARG00000017107) |  | NR2E1  (ENSG00000112333) |  |
| **GO term name** | **GO domain** | **GO term name** | **GO domain** |
| DNA binding | molecular_function | DNA binding | molecular_function |
| DNA binding transcription factor activity | molecular_function | DNA binding transcription factor activity | molecular_function |
| metal ion binding | molecular_function | enzyme binding | molecular_function |
| sequence-specific DNA binding | molecular_function | histone deacetylase binding | molecular_function |
| steroid hormone receptor activity | molecular_function | metal ion binding | molecular_function |
| zinc ion binding | molecular_function | RNA polymerase II transcription factor activity, sequence-specific DNA binding | molecular_function |
| nucleus | cellular_component | sequence-specific DNA binding | molecular_function |
| regulation of transcription, DNA-templated | biological_process | steroid hormone receptor activity | molecular_function |
| steroid hormone mediated signaling pathway | biological_process | transcriptional activator activity, RNA polymerase II proximal promoter sequence-specific DNA binding | molecular_function |
| transcription, DNA-templated | biological_process | transcriptional repressor activity, RNA polymerase II proximal promoter sequence-specific DNA binding | molecular_function |
|  |  | zinc ion binding | molecular_function |
|  |  | nucleoplasm | cellular_component |
|  |  | nucleus | cellular_component |
|  |  | aggressive behavior | biological_process |
|  |  | amygdala development | biological_process |
|  |  | anterior commissure morphogenesis | biological_process |
|  |  | behavioral fear response | biological_process |
|  |  | brain development | biological_process |
|  |  | camera-type eye development | biological_process |
|  |  | cell fate commitment | biological_process |
|  |  | cerebral cortex development | biological_process |
|  |  | cerebral cortex neuron differentiation | biological_process |
|  |  | dentate gyrus development | biological_process |
|  |  | extracellular matrix organization | biological_process |
|  |  | forebrain generation of neurons | biological_process |
|  |  | glial cell migration | biological_process |
|  |  | layer formation in cerebral cortex | biological_process |
|  |  | long-term synaptic potentiation | biological_process |
|  |  | multicellular organism development | biological_process |
|  |  | negative regulation of apoptotic process | biological_process |
|  |  | negative regulation of astrocyte differentiation | biological_process |
|  |  | negative regulation of neural precursor cell proliferation | biological_process |
|  |  | negative regulation of neuron differentiation | biological_process |
|  |  | negative regulation of transcription from RNA polymerase II promoter | biological_process |
|  |  | nervous system development | biological_process |
|  |  | olfactory bulb development | biological_process |
|  |  | positive regulation of angiogenesis | biological_process |
|  |  | positive regulation of cell cycle | biological_process |
|  |  | positive regulation of cell proliferation | biological_process |
|  |  | positive regulation of neural precursor cell proliferation | biological_process |
|  |  | positive regulation of stem cell proliferation | biological_process |
|  |  | positive regulation of transcription from RNA polymerase II promoter | biological_process |
|  |  | regulation of cell migration involved in sprouting angiogenesis | biological_process |
|  |  | regulation of cellular component organization | biological_process |
|  |  | regulation of dendrite morphogenesis | biological_process |
|  |  | regulation of timing of neuron differentiation | biological_process |
|  |  | regulation of transcription, DNA-templated | biological_process |
|  |  | retina development in camera-type eye | biological_process |
|  |  | social behavior | biological_process |
|  |  | somatic stem cell population maintenance | biological_process |
|  |  | steroid hormone mediated signaling pathway | biological_process |
|  |  | transcription initiation from RNA polymerase II promoter | biological_process |
|  |  | transcription, DNA-templated | biological_process |
|  |  | visual perception | biological_process |
