# Supplementary material for: Evolutionarily novel genes are expressed in transgenic fish tumors and their orthologs are involved in development of progressive traits in humans
Source: Infect Agent Cancer. 2019 Dec 5;14:46. doi: 10.1186/s13027-019-0262-5 (PMC6896781; doi:10.1186/s13027-019-0262-5)
Supplement: Supplementary file 21 — Additional file 21. GO annotation of fish TSEEN mycn and it’s human ortholog MYCN [file 13027_2019_262_MOESM21_ESM.doc]

Table – GO annotation of fish TSEEN mycn and it’s human ortholog MYCN

| *Danio rerio* | | *Homo sapiens* | |
| --- | --- | --- | --- |
| *mycn* (ENSDARG00000006837) |  | *MYCN*  (ENSG00000134323) |  |
| **GO term name** | **GO domain** | **GO term name** | **GO domain** |
| DNA binding transcription factor activity | molecular_function | DNA binding | molecular_function |
| protein dimerization activity | molecular_function | DNA binding transcription factor activity | molecular_function |
| nucleus | cellular_component | kinase binding | molecular_function |
| regulation of transcription, DNA-templated | biological_process | protein binding | molecular_function |
|  |  | protein dimerization activity | molecular_function |
|  |  | RNA polymerase II proximal promoter sequence-specific DNA binding | molecular_function |
|  |  | RNA polymerase II transcription factor activity, sequence-specific DNA binding | molecular_function |
|  |  | transcriptional activator activity, RNA polymerase II proximal promoter sequence-specific DNA binding | molecular_function |
|  |  | chromatin | cellular_component |
|  |  | nucleolus | cellular_component |
|  |  | nucleus | cellular_component |
|  |  | branching morphogenesis of an epithelial tube | biological_process |
|  |  | cartilage condensation | biological_process |
|  |  | embryonic digit morphogenesis | biological_process |
|  |  | embryonic skeletal system morphogenesis | biological_process |
|  |  | lung development | biological_process |
|  |  | negative regulation of astrocyte differentiation | biological_process |
|  |  | negative regulation of gene expression | biological_process |
|  |  | negative regulation of reactive oxygen species metabolic process | biological_process |
|  |  | positive regulation of cell death | biological_process |
|  |  | positive regulation of cell proliferation | biological_process |
|  |  | positive regulation of gene expression | biological_process |
|  |  | positive regulation of mesenchymal cell proliferation | biological_process |
|  |  | positive regulation of production of miRNAs involved in gene silencing by miRNA | biological_process |
|  |  | positive regulation of transcription from RNA polymerase II promoter | biological_process |
|  |  | positive regulation of transcription, DNA-templated | biological_process |
|  |  | regulation of inner ear auditory receptor cell differentiation | biological_process |
|  |  | regulation of transcription from RNA polymerase II promoter | biological_process |
|  |  | regulation of transcription, DNA-templated | biological_process |
|  |  | transcription from RNA polymerase II promoter | biological_process |
|  |  | transcription, DNA-templated | biological_process |
