# Supplementary material for: Evolutionarily novel genes are expressed in transgenic fish tumors and their orthologs are involved in development of progressive traits in humans
Source: Infect Agent Cancer. 2019 Dec 5;14:46. doi: 10.1186/s13027-019-0262-5 (PMC6896781; doi:10.1186/s13027-019-0262-5)
Supplement: Supplementary file 22 — Additional file 22. GO annotation of fish TSEEN fosl1a and it’s human ortholog FOSL1. [file 13027_2019_262_MOESM22_ESM.doc]

**Table – GO annotation of fish TSEEN fosl1a and it’s human ortholog FOSL1**

| FOS-like antigen 1a (fosl1a) |  | FOS-like antigen 1 (FOSL1) |  |
| --- | --- | --- | --- |
| *Danio rerio* |  | *Human* |  |
| **Gene stable ID** | **Gene name** | **Gene stable ID** | **Gene name** |
| ENSDARG00000015355 | *fosl1a* | ENSG00000175592 | *FOSL1* |
|  |  |  |  |
| **GO term name** | **GO domain** | **GO term name** | **GO domain** |
| DNA binding | molecular_function | DNA binding | molecular_function |
| DNA binding transcription factor activity | molecular_function | DNA binding transcription factor activity | molecular_function |
| regulation of transcription, DNA-templated | biological_process | protein binding | molecular_function |
| regulation of transcription from RNA polymerase II promoter | biological_process | RNA polymerase II proximal promoter sequence-specific DNA binding | molecular_function |
|  |  | RNA polymerase II regulatory region sequence-specific DNA binding | molecular_function |
|  |  | RNA polymerase II transcription factor activity, sequence-specific DNA binding | molecular_function |
|  |  | transcriptional activator activity, RNA polymerase II proximal promoter sequence-specific DNA binding | molecular_function |
|  |  | cytosol | cellular_component |
|  |  | intracellular membrane-bounded organelle | cellular_component |
|  |  | neuron projection | cellular_component |
|  |  | nucleoplasm | cellular_component |
|  |  | nucleus | cellular_component |
|  |  | presynaptic membrane | cellular_component |
|  |  | cellular defense response | biological_process |
|  |  | cellular response to extracellular stimulus | biological_process |
|  |  | chemotaxis | biological_process |
|  |  | female pregnancy | biological_process |
|  |  | in utero embryonic development | biological_process |
|  |  | learning | biological_process |
|  |  | negative regulation of cell proliferation | biological_process |
|  |  | placenta blood vessel development | biological_process |
|  |  | positive regulation of apoptotic process | biological_process |
|  |  | positive regulation of cell cycle | biological_process |
|  |  | positive regulation of cell proliferation | biological_process |
|  |  | positive regulation of DNA binding transcription factor activity | biological_process |
|  |  | positive regulation of DNA-templated transcription, initiation | biological_process |
|  |  | positive regulation of transcription from RNA polymerase II promoter | biological_process |
|  |  | pri-miRNA transcription from RNA polymerase II promoter | biological_process |
|  |  | regulation of transcription from RNA polymerase II promoter | biological_process |
|  |  | regulation of transcription, DNA-templated | biological_process |
|  |  | response to cAMP | biological_process |
|  |  | response to corticosterone | biological_process |
|  |  | response to cytokine | biological_process |
|  |  | response to drug | biological_process |
|  |  | response to gravity | biological_process |
|  |  | response to hydrogen peroxide | biological_process |
|  |  | response to mechanical stimulus | biological_process |
|  |  | response to organic cyclic compound | biological_process |
|  |  | response to progesterone | biological_process |
|  |  | response to virus | biological_process |
|  |  | transcription from RNA polymerase II promoter | biological_process |
|  |  | vitellogenesis | biological_process |
