# Supplementary material for: Evolutionarily novel genes are expressed in transgenic fish tumors and their orthologs are involved in development of progressive traits in humans
Source: Infect Agent Cancer. 2019 Dec 5;14:46. doi: 10.1186/s13027-019-0262-5 (PMC6896781; doi:10.1186/s13027-019-0262-5)
Supplement: Supplementary file 23 — Additional file 23. Source code of Original scripts “Alignment BLAST”, (BLAST database creation, Fasta slasher, OMA parameters). [file 13027_2019_262_MOESM23_ESM.docx]

Source code of Original scripts “Alignment BLAST”, (BLAST database creation, Fasta slasher, OMA parameters)

Alignment BLAST

#!/bin/sh

#DB='DB_list.txt'

g=`find /media/andrew/49a47684-4a07-4f13-b9bb-930c306d951f/Danio/Genes/Lost_genes -name "*fasta"`

mkdir /media/andrew/49a47684-4a07-4f13-b9bb-930c306d951f/Danio/Genes/Completed/

folder=/media/andrew/49a47684-4a07-4f13-b9bb-930c306d951f/Danio/Genes/Aligned/

mkdir -p $folder

for gen in $g

do

echo "Processing $gen"

gen_c=`echo ${gen} | sed 's/.*Lost_genes\///' | sed -e 's/.fasta//'`

#folder=/media/andrew/49a47684-4a07-4f13-b9bb-930c306d951f/Danio/Genes/${gen_c}

#mkdir -p $folder

#for genome in $f

#do

DB='/media/andrew/49a47684-4a07-4f13-b9bb-930c306d951f/Cancer_genes/Oncogenes/Genomes/BLAST_DB/db.txt'

while read GENOME; do

echo "Aligning $gen_c $GENOME"

#genome_name=`echo $GENOME | sed -e 's/.db//' | sed "s/.*Genomes_hmmer_np\///"`

#out=`echo $genome`

#out=${folder}/${GENOME}_${gen_c}_tab.out

outn=${folder}/${GENOME}_${gen_c}.out

blastn -db $GENOME -query $gen -out $outn

done < $DB

echo "$gene_c finished"

mv ${gen} /media/andrew/49a47684-4a07-4f13-b9bb-930c306d951f/Danio/Genes/Completed/

done

#DB='/media/andrew/49a47684-4a07-4f13-b9bb-930c306d951f/Danio/236_shuf.txt'

#while read GENOME; do

# echo "Это строка: $GENOME"

#done < $DB

#echo "Hi!"

BLAST database creation

#dustmasker -in Ciona.faa -infmt fasta -parse_seqids -outfmt maskinfo_asn1_bin -out Ciona_dust.asnb

segmasker -in Carp.faa -infmt fasta -parse_seqids -outfmt maskinfo_asn1_bin -out Carp.asnb

#makeblastdb -in Ciona.faa -input_type fasta -dbtype prot -parse_seqids -mask_data Ciona_dust.asnb -out Carp

makeblastdb -in Carp.faa -input_type fasta -dbtype prot -parse_seqids -mask_data Carp.asnb -out Carp -title "Carp"

blastdbcmd -db Ciona -info

Fasta slasher

#!/bin/sh

#mkdir /media/andrew/49a47684-4a07-4f13-b9bb-930c306d951f/Danio/Genes/Lost_genes/

LIST='/media/andrew/49a47684-4a07-4f13-b9bb-930c306d951f/Danio/Genes/names.txt'

while read NAME; do

echo "Extracting $NAME"

folder="/media/andrew/49a47684-4a07-4f13-b9bb-930c306d951f/Danio/Genes/Lost_genes/"

out=${folder}/${NAME}.fasta

samtools faidx 149_missed_genes.fasta $NAME > $out

echo "$NAME finished"

done < $LIST

OMA parameters:

InputDataType := 'AA';

OutputFolder := 'Output';

ReuseCachedResults := true;

AlignBatchSize := 1e6;

MinScore := 181;

LengthTol := 0.61;

StablePairTol := 1.81;

InparalogTol := 3.00;

ParalogTol := -2.5*StablePairTol;

VerifiedPairTol := 1.53;

MinSeqLen := 50;

UseOnlyOneSplicingVariant := true;

UseExperimentalHomologousClusters := false;

QuasiCliquesCutoff := 1.0:

StableIdsForGroups := false;

GuessIdType := false;

DoHierarchicalGroups := 'top-down';

MaxTimePerLevel := 1200;

SpeciesTree := 'estimate';

ReachabilityCutoff := 0.65;

MinEdgeCompletenessFraction := 0.8;
